# Angiopoietin-2 blockade ameliorates autoimmune neuroinflammation by inhibiting leukocyte recruitment into the CNS

**DOI:** 10.1172/JCI130308

**Published:** 2020-03-09

**Authors:** Zhilin Li, Emilia A. Korhonen, Arianna Merlini, Judith Strauss, Eleonoora Wihuri, Harri Nurmi, Salli Antila, Jennifer Paech, Urban Deutsch, Britta Engelhardt, Sudhakar Chintharlapalli, Gou Young Koh, Alexander Flügel, Kari Alitalo

**Affiliations:** 1 Wihuri Research Institute and Translational Cancer Medicine Program, Faculty of Medicine, University of Helsinki, Helsinki, Finland.; 2 Institute for Neuroimmunology and Multiple Sclerosis Research, University Medical Centre Göttingen, Göttingen, Germany.; 3 Theodor Kocher Institute, University of Bern, Bern, Switzerland.; 4 Oncology Research, Lilly Research Laboratories, Eli Lilly and Co., Indianapolis, Indiana, USA.; 5 Center for Vascular Research, Institute for Basic Science, Daejeon, South Korea.; 6 Graduate School of Medical Science and Engineering, Korea Advanced Institute of Science and Technology (KAIST), Daejeon, South Korea.; 7 Helsinki Institute of Life Science, University of Helsinki, Helsinki, Finland.

**Keywords:** Autoimmunity, Neurological disorders

## Abstract

Angiopoietin-2 (Ang2), a ligand of the endothelial Tie2 tyrosine kinase, is involved in vascular inflammation and leakage in critically ill patients. However, the role of Ang2 in demyelinating central nervous system (CNS) autoimmune diseases is unknown. Here, we report that Ang2 is critically involved in the pathogenesis of experimental autoimmune encephalomyelitis (EAE), a rodent model of multiple sclerosis. Ang2 expression was induced in CNS autoimmunity, and transgenic mice overexpressing Ang2 specifically in endothelial cells (ECs) developed a significantly more severe EAE. In contrast, treatment with Ang2-blocking Abs ameliorated neuroinflammation and decreased spinal cord demyelination and leukocyte infiltration into the CNS. Similarly, Ang2-binding and Tie2-activating Ab attenuated the development of CNS autoimmune disease. Ang2 blockade inhibited expression of EC adhesion molecules, improved blood-brain barrier integrity, and decreased expression of genes involved in antigen presentation and proinflammatory responses of microglia and macrophages, which was accompanied by inhibition of α_5_β_1_ integrin activation in microglia. Taken together, our data suggest that Ang2 provides a target for increasing Tie2 activation in ECs and inhibiting proinflammatory polarization of CNS myeloid cells via α_5_β_1_ integrin in neuroinflammation. Thus, Ang2 targeting may serve as a therapeutic option for the treatment of CNS autoimmune disease.

## Graphical abstract

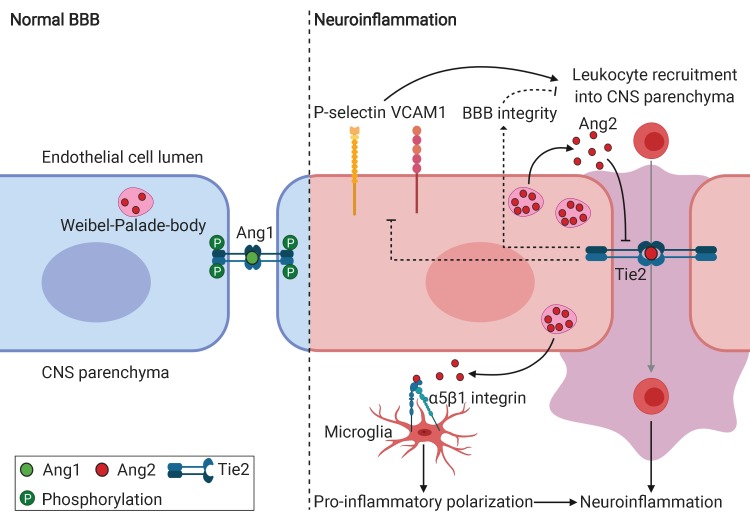

## Introduction

The blood-brain barrier (BBB) is indispensable for the maintenance of CNS homeostasis, acting by restricting molecular and cellular trafficking across the blood vascular endothelium into the CNS (1). Compromised BBB integrity and leukocyte infiltration into the CNS are crucial in the pathogenesis of inflammatory CNS diseases (2). Immune cell activation leads to demyelination and axonal damage, resulting in neurological dysfunction and physical disability (3). Thus, identifying novel targets for therapeutic intervention that can improve BBB integrity and attenuate leukocyte recruitment into the CNS holds great promise for the development of effective strategies for treating CNS autoimmune diseases. Experimental autoimmune encephalomyelitis (EAE) is a well-characterized experimental model of multiple sclerosis (MS), which is the most common human demyelinating CNS autoimmune disease (3). This makes EAE an ideal model for studying the cellular and molecular mechanisms underlying CNS autoimmune disease.

During inflammation, endothelial cells (ECs) are critically involved in regulating vascular permeability and leukocyte recruitment. The angiopoietin-Tie (Ang-Tie) growth factor–receptor system is essential for vascular development and integrity and for vessel remodeling in pathological conditions (4, 5). The Ang ligands bind to and regulate activation of the Tie1-Tie2 receptor tyrosine kinase complex. Ang1 is a constitutively expressed agonistic ligand that promotes vascular stability, whereas Ang2 is a context-dependent antagonist or agonist of Tie2 (6–9). Overexpression of an Ang2 transgene in ECs has been shown to increase BBB permeability for low molecular weight solutes in vivo (10). In inflammatory conditions, Ang2 is induced in the ECs, in which it promotes vascular remodeling and leakage by antagonizing phosphorylation of the constitutively active endothelial Tie2 tyrosine kinase receptor and by activating α_5_β_1_ integrin (11–15). The unique role of the Ang/Tie signaling pathway in vascular stability suggests that it could serve as a target of therapeutic intervention in diseases in which vascular integrity is compromised (4). Although Ang2 has been implicated in cancer, myocardial ischemia, and sepsis (11, 16–18), its possible involvement in CNS autoimmune disease remains unknown.

Here we explored the role of Ang2 in autoimmune neuroinflammation by using mice expressing an Ang2 transgene specifically in ECs and a function-blocking Ab against Ang2. Our results show that Ang2 is induced in neuroinflammation and that Ang2 overexpression in ECs exacerbates the development of CNS autoimmune disease. Mechanistically, we demonstrate that Ang2 upregulates the neuroinflammation-induced expression of EC adhesion molecules and exacerbates BBB permeability, thereby enhancing leukocyte recruitment, and promotes proinflammatory polarization and responses of myeloid cells in the CNS. Hence, our study identifies Ang2 as a potential target for therapeutic intervention. The Ang2-blocking Ab that attenuates both vascular and neuroinflammation should provide a possibility for the treatment of CNS autoimmune diseases.

## Results

### Ang2 overexpression exacerbates whereas its blockade ameliorates the severity of CNS autoimmune disease.

**Figure 1:**
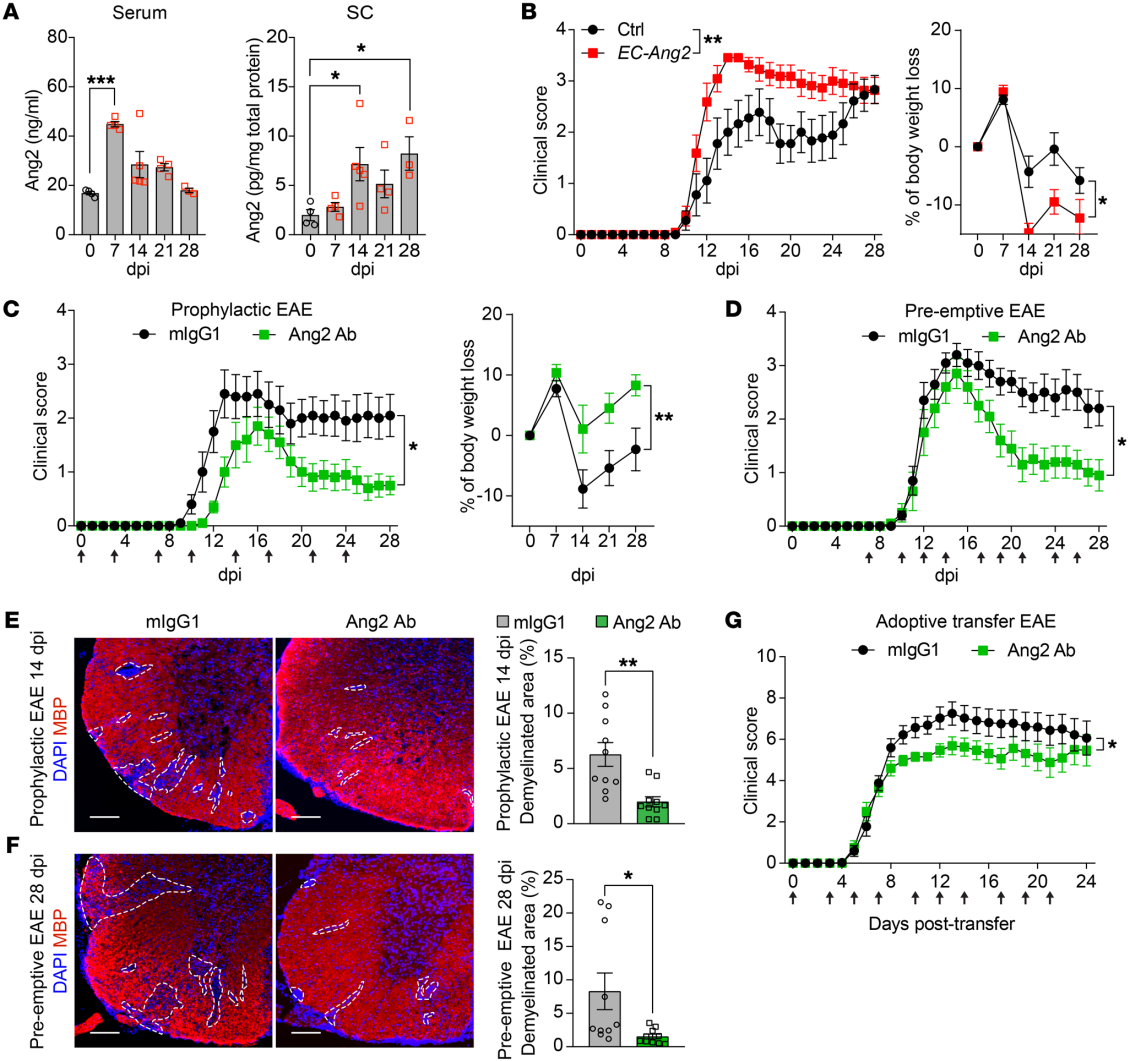
Ang2 is induced in EAE, and Ang2 blockade ameliorates EAE. (**A**) Ang2 protein concentration in the serum and SC lysates at different time points after EAE induction (0 dpi: *n* = 4; 7 dpi: *n* = 4; 14 dpi: *n* = 5; 21 dpi: *n* = 4; 28 dpi: *n* = 3). (**B**) Clinical scores and percentage of body weight loss of control (Ctrl, *n* = 9) versus *EC-Ang2* (*n* = 11) mice induced with active EAE. (**C**) Clinical scores and percentages of body weight loss of mice induced with active EAE and treated with mIgG1 versus Ang2 Ab prophylactically (starting at the time of EAE induction; 0 dpi) (*n* = 10 per group). (**D**) Clinical scores of mice induced with active EAE and treated with mIgG1 versus Ang2 Ab preemptively (starting during the effector phase of EAE at 7 dpi) (*n* = 10 per group). (**E** and **F**) Representative images and quantifications of MBP staining to show loss of myelin in the SC white matter from both prophylactic 14 dpi and preemptive 28 dpi groups (*n* = 10 per group). Scale bars: 100 μm. (**G**) Clinical scores of mice induced with adoptive transfer EAE and treated with mIgG1 versus Ang2 Ab starting at the time of adoptive transfer. Data are pooled from 2 independent experiments (*n* = 16 per group). Arrows indicate Ab injections. Mean ± SEM, 1-way ANOVA with Dunnett’s post hoc test for multiple comparisons (**A**), nonparametric Mann-Whitney *U* test (**B**-**D**, and **G**, comparison of AUC values of clinical EAE scores over the disease course), 2-way repeated measures ANOVA (**B** and **C**, body weight loss), and 2-tailed Student’s *t* test (**E** and **F**). *P < 0.05; **P < 0.01; ***P < 0.001.

In order to investigate the possible involvement of Ang2 in autoimmune inflammation of the CNS, we first measured Ang2 protein concentrations in serum and spinal cord (SC) lysates of adult C57BL/6J mice before and at different time points after immunization with myelin oligodendrocyte glycoprotein (MOG)_35–55_ peptide (active EAE). As shown in Figure 1A, Ang2 protein concentrations in the serum at 7 days post immunization (dpi) and in the SCs at 14 and 28 dpi were higher than in naive mice (0 dpi), indicating induction of Ang2 expression during CNS autoimmune disease development.

We next determined whether an excess of Ang2 would influence the autoimmune process within the CNS. We induced active EAE in mice that overexpress a tetracycline-regulated Ang2 transgene specifically in the ECs (*VE-cadherin-tTA*;*Tet-OS-Ang2* double transgenic, designated as *EC-Ang2* mice) (17). Interestingly, these mice developed a more severe disease, as characterized by higher EAE clinical scores and exacerbated body weight loss (Figure 1B). To ascertain whether blocking Ang2 function ameliorates neuroinflammation, we first treated mice with mouse IgG1 (mIgG1) isotype control or Ang2-blocking Ab (Ang2 Ab) starting at the time of EAE induction (0 dpi). The prophylactic Ang2 blockade attenuated the clinical severity of the disease and body weight loss when compared with mIgG1-treated control mice (Figure 1C). We then assessed the potential therapeutic effect of the Ang2 Ab in neuroinflammation by administrating the Abs starting immediately before EAE onset (at 7 dpi), during the effector phase of the disease (preemptive EAE). Again, Ang2 blockade, but not application of mIgG1, resulted in reduction of disease severity (Figure 1D). Moreover, Ang2 Ab–treated EAE mice had smaller demyelinated lesion areas in the SCs than mIgG1-treated control mice, as demonstrated by immunostaining of myelin basic protein (MBP) (Figure 1, E and F). These data showed that Ang2 is critically involved in CNS autoimmune pathogenesis.

Results from the preemptive Ang2 blockade indicated that Ang2 exerts a more important pathogenic role during the effector phase of the CNS autoimmune process, rather than during the T cell priming phase in the peripheral organs. In order to confirm this, we induced EAE in mice by adoptive transfer of fully differentiated, fluorescently labeled effector T cells (adoptive transfer EAE) and found that Ang2 Ab treatment also exerted a significant therapeutic effect (Figure 1G). Intravital 2-photon laser scanning microscopy of EAE lesions revealed that Ang2 blockade did not affect the basic locomotion characteristics of the fluorescent effector T cells in the SCs, as neither the speed, movement displacement, nor straightness of T cells was changed (Supplemental Figure 1A; supplemental material available online with this article; https://doi.org/10.1172/JCI130308DS1). However, even in mice at a similar disease stage, Ang2 blockade led to a reduction of the number of infiltrated effector T cells (Supplemental Figure 1B), which suggests that Ang2 is involved in the regulation of immune cell recruitment into the CNS.

### Ang2 blockade attenuates leukocyte recruitment into the CNS.

**Figure 2:**
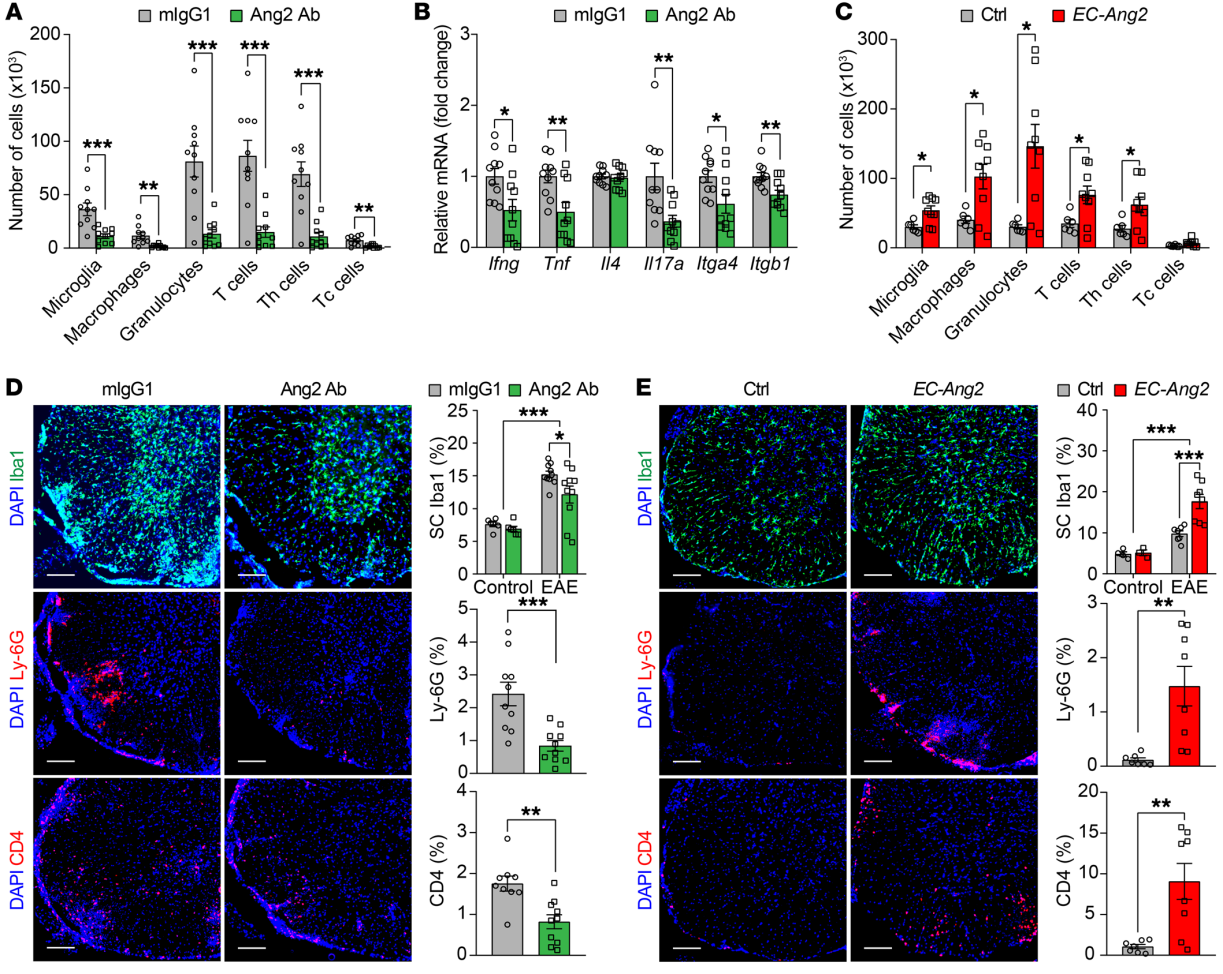
Prophylactic Ang2 blockade attenuates leukocyte infiltration and inflammation in the SCs of EAE mice. (**A**) Flow cytometric quantification of the number of immune cells in the SCs of mIgG1- versus Ang2 Ab–treated EAE mice at 12 dpi (*n* = 10 per group). (**B**) RT-qPCR quantification of mRNA levels of Th signature cytokines (*Ifng*, *Tnf*, *Il4*, and *Il17a*) and integrin subunits (*Itga4* and *Itgb1*) in the SCs of mIgG1- versus Ang2 Ab–treated EAE mice at 14 dpi (*n* = 10 per group). (**C**) Flow cytometric quantification of the number of immune cells in the SCs of control (*n* = 6) versus *EC-Ang2* (*n* = 9) EAE mice at 12 dpi. (**D** and **E**) Representative immunofluorescent images and quantifications of Iba1^+^ microglia and macrophages, Ly-6G^+^ granulocytes, and CD4^+^ Th cells in the SCs of mIgG1- versus Ang2 Ab–treated control and EAE mice (*n* = 10 per group) at 14 dpi as well as in the SCs of control (*n* = 7) versus *EC-Ang2* (*n* = 8) control and EAE mice at 12 dpi. Scale bars: 100 μm. Mean ± SEM, 2-tailed Student’s *t* test (**A**-**E**), and 2-way ANOVA with Bonferroni’s post hoc test for multiple comparisons (Iba1 staining in **D** and **E**). *P < 0.05; **P < 0.01; ***P < 0.001.

To analyze the extent of leukocyte infiltration into the CNS, we enriched immune cells from the SCs of mIgG1- and Ang2 Ab–treated EAE mice at 12 dpi by Percoll gradient centrifugation (19). Flow cytometric quantification showed that microglia, macrophages, granulocytes, and T cells (both Th cells and cytotoxic T [Tc] cells) were decreased in the SCs of Ang2 Ab–treated EAE mice in comparison with mIgG1-treated control mice (Figure 2A and Supplemental Figure 2A). In addition, we quantified gene expression of classical Th1 (*Ifng* and *Tnf*), Th2 (*Il4*), and Th17 cytokines (*Il17a*) and found that the Ang2 Ab–treated EAE mice expressed lower mRNA levels of proinflammatory Th1 and Th17 cytokines in the SCs than mIgG1-treated control mice at 14 dpi (Figure 2B). Moreover, we detected downregulation of *Itga4* and *Itgb1* subunits of α_4_β_1_ integrin, which is involved in T cell trafficking, in the SCs of the Ang2 Ab–treated EAE mice (Figure 2B). In contrast, flow cytometric analysis revealed that the *EC-Ang2* EAE mice had more microglia, macrophages, granulocytes, and T cells (Th cells) in the SCs than single transgenic control mice at 12 dpi (Figure 2C). These flow cytometric data were further consolidated by immunohistochemical staining of SC sections using Abs against the microglia and macrophage marker Iba1, granulocyte marker Ly-6G, and Th cell marker CD4 (Figure 2, D and E). However, neither Ang2 blockade nor Ang2 overexpression affected the number of Iba1^+^ cells in the SCs of control mice (Figure 2, D and E), suggesting that Ang2 does not influence microglial activity during homeostasis. These results show that Ang2 promotes leukocyte infiltration into the CNS in neuroinflammation without affecting the number of microglia under physiological conditions.

### Ang2 blockade ameliorates immune cell activation and restores CNS homeostasis.

**Figure 3:**
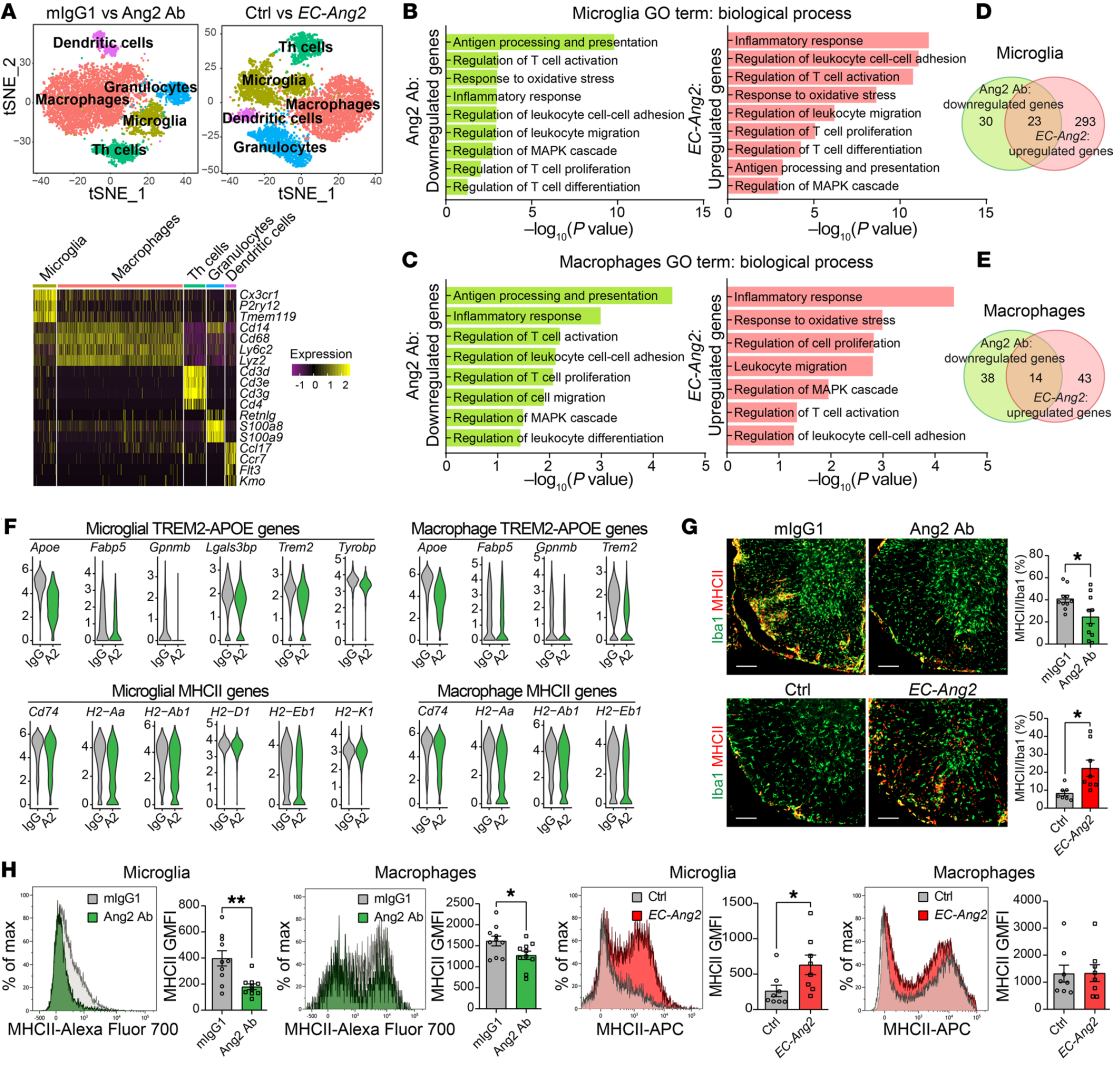
Prophylactic Ang2 blockade dampens inflammatory responses of immune cells in the SCs of EAE mice. (**A**) T-distributed stochastic neighbor embedding (t-SNE) analysis of main immune cell clusters in the SCs of mIgG1- versus Ang2 Ab– treated EAE mice at 14 dpi and single transgenic control versus *EC-Ang2* EAE mice at 12 dpi. Heatmap showing log_2_ expression of known marker genes for each cluster. (**B** and **C**) Relevant GO biological processes of significantly (adjusted *P* < 0.05) downregulated genes after Ang2 blockade and upregulated genes after Ang2 overexpression in microglia (**B**) and macrophages (**C**). (**D** and **E**) Venn diagram illustrating the number of genes that were regulated by Ang2 in microglia and macrophages. (**F**) Violin plots showing mRNA expression of significantly (adjusted *P* < 0.05) downregulated APOE-induced and MHCII-associated genes after Ang2 blockade. IgG, mIgG1; A2, Ang2 Ab. (**G**) Representative images and quantifications of MHCII immunostaining in Iba1^+^ cells in the SCs of mIgG1- versus Ang2 Ab–treated EAE mice at 14 dpi (*n* = 10 per group) and control (*n* = 7) versus *EC-Ang2* (*n* = 8) EAE mice at 12 dpi. Scale bars: 100 μm. (**H**) Representative flow cytometry overlay plots and quantifications showing GMFI of MHCII expression in microglia and macrophages in the SCs of mIgG1- versus Ang2 Ab–treated EAE mice at 14 dpi (*n* = 10 per group) and control versus *EC-Ang2* EAE mice at 12 dpi (*n* = 8 per group). Mean ± SEM, 2-tailed Student’s *t* test (**G** and **H**). *P < 0.05; **P < 0.01.

To map the profiles of immune cells, we sorted CD45^+^ immune cells from the SCs of mIgG1- versus Ang2 Ab–treated EAE mice at 14 dpi and single transgenic control versus *EC-Ang2* EAE mice at 12 dpi (Supplemental Figure 2B) and analyzed their transcriptomes by single-cell RNA-Seq (scRNA-Seq). Hierarchical clustering of the cells identified 5 main clusters of immune cells (Figure 3A), which expressed canonical markers of microglia (*Tmem119*, *P2ry12*, and *Cx3cr1*), macrophages (*Cd14*, *Lyz2*, *Ly6c2*, and *Cd68*), Th cells (*Cd3d*, *Cd3e*, *Cd3g*, and *Cd4*), granulocytes (*Retnlg*, *S100a8*, and *S100a9*), and dendritic cells (*Kmo*, *Flt3*, *Ccr7*, and *Ccl17*) (Figure 3A). The largest number of significantly (adjusted *P* < 0.05) downregulated genes by Ang2 blockade was found in microglia, followed by macrophages, granulocytes, Th cells, and dendritic cells. Gene ontology (GO) biological process analysis of the differentially expressed genes revealed that the downregulated genes in the microglial cluster were involved in antigen processing and presentation, inflammatory response, regulation of leukocyte cell-cell adhesion, regulation of leukocyte migration, regulation of T cell activation, proliferation and differentiation, and regulation of MAPK cascade (Figure 3B). In addition, Ang2 blockade decreased the expression of genes involved in the response to oxidative stress that is associated with neurotoxicity (Figure 3B) (20), indicating that Ang2 blockade exerts neuroprotective effects during neuroinflammation. Similar biological processes were also downregulated in the macrophage cluster (Figure 3C). In contrast, most of the GO biological processes downregulated in the microglial and macrophage clusters of the Ang2 Ab–treated EAE mice were upregulated in the corresponding clusters of the *EC-Ang2* EAE mice (Figure 3, B and C).

Altogether, 23 genes in the microglia and 14 genes in the macrophages were inversely regulated by Ang2 blockade and overexpression (Figure 3, D and E, and Supplemental Table 1). Notably, the TREM2/APOE pathway (*Apoe*, *Trem2*, *Fabp5*, *Gpnmb*, and *Tyrobp*), which is a major regulator driving microglial transition from a homeostatic to a neurodegenerative/neuroinflammatory phenotype in neurodegenerative and autoimmune diseases (Alzheimer’s disease, amyotrophic lateral sclerosis, and MS) (21), was also downregulated in the microglial and macrophage clusters by Ang2 blockade (Figure 3F), which was confirmed by quantitative reverse-transcription PCR (RT-qPCR) (Supplemental Figure 2C). However, Ang2 blockade did not alter the expression of homeostatic genes or transcription factors in microglia or macrophages (Supplemental Figure 2, D and E). Decreased expression of MHCII-associated genes (*H2-Aa*, *H2-Ab1*, *H2-Eb1*, *H2-K1*, and *H2-Q7*) revealed that Ang2 blockade dampens the proinflammatory phenotype of microglia and macrophages (Figure 3F). These results were validated by RT-qPCR, flow cytometry, and immunostaining of MHCII in Iba1^+^ cells (Figure 3, G and H, and Supplemental Figure 2C). In contrast, Ang2 overexpression in ECs led to upregulation of APOE-induced genes and MHCII in CNS myeloid cells (Figure 3, G and H, and Supplemental Figure 2, F and G).

### Ang2 blockade inhibits proinflammatory polarization of microglia by dampening α_5_β_1_ integrin activation.

**Figure 4:**
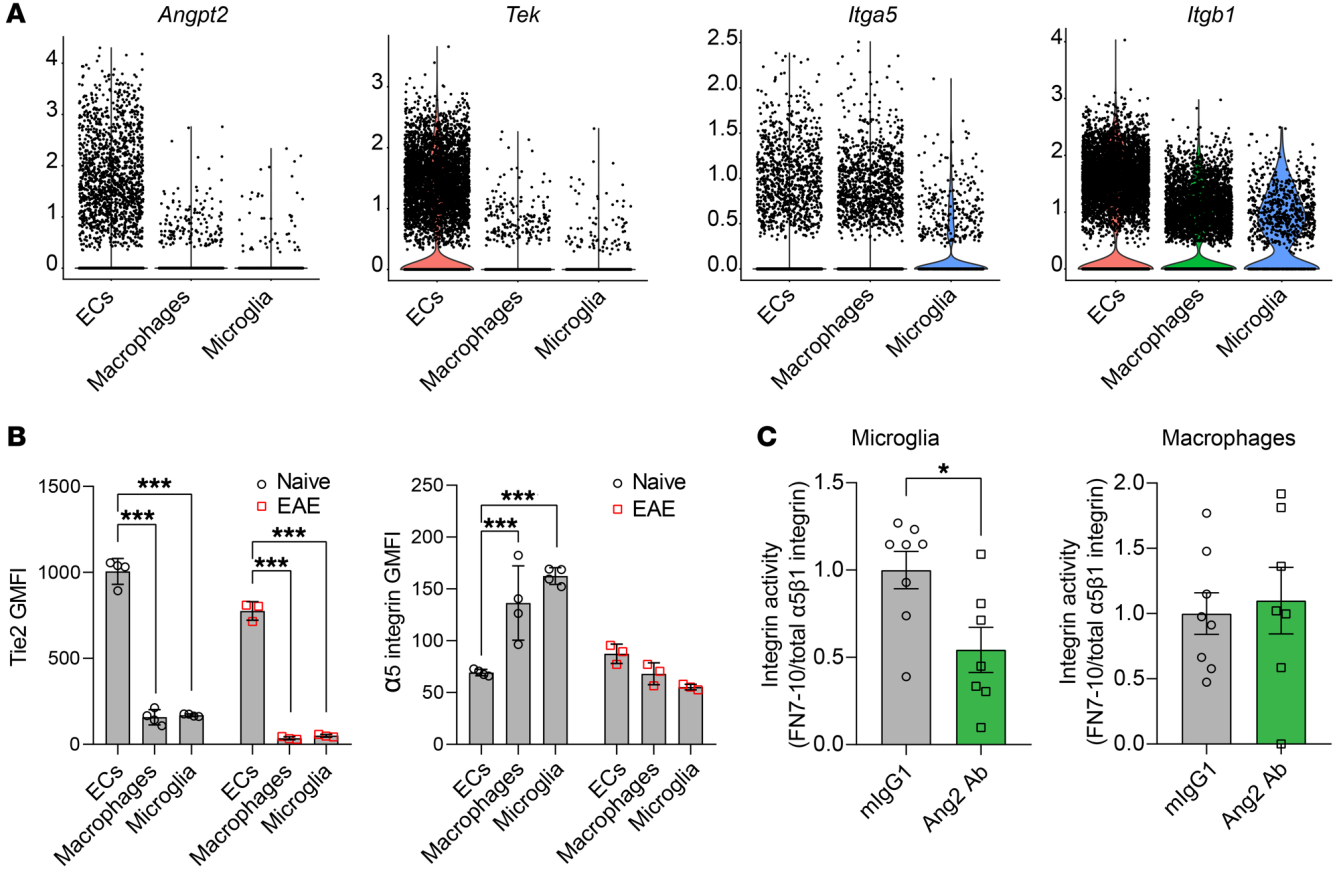
Prophylactic Ang2 blockade inhibits α_5_β_1_ integrin activation in the SCs of EAE mice. (**A**) Violin plots showing expression of *Angpt2* and its receptors *Tek*, *Itga5*, and *Itgb1* in EC, microglia, and macrophage clusters of mIgG1- and Ang2 Ab–treated EAE mice. (**B**) GMFI of Tie2 and α_5_ integrin on the surface of ECs, macrophages, and microglia from naive versus EAE mice (naive, *n* = 4; EAE, *n* = 3) at 14 dpi as analyzed by flow cytometry. (**C**) Flow cytometric analysis of active cell surface integrin (FN7-10 binding) relative to total cell surface α_5_β_1_ integrin in microglia and macrophages from mIgG1- and Ang2 Ab–treated EAE mice (mIgG1, *n* = 8; Ang2 Ab, *n* = 7) at the disease peak (16 dpi). Mean ± SEM, 2-way ANOVA with Bonferroni’s post hoc test for multiple comparisons (**B**) and 2-tailed Student’s *t* test (**C**). *P < 0.05; ***P < 0.001.

In the inflamed SC, ECs, in contrast with macrophages and microglia, expressed high levels of *Angpt2* and *Tek* (Figure 4A). However, ECs, macrophages, and microglia expressed comparable levels of subunits of α_5_β_1_ integrin (Figure 4A), which is activated by Ang2 (11, 22). To validate our scRNA-Seq data, we enriched ECs and immune cells from the SCs of both naive and EAE mice at 14 dpi and analyzed their surface expression of Tie2 and α_5_ integrin by flow cytometry. The result showed that macrophages and microglia express levels of α_5_ integrin similar to those of ECs, but much less Tie2 (Figure 4B), suggesting that α_5_β_1_ integrin is the main receptor that mediates Ang2 function in macrophages and microglia. In order to test this hypothesis, we used flow cytometry to analyze the binding of macrophages and microglia to fluorescently labeled fibronectin type III repeats 7-10 (FN7-10), which binds primarily to α_5_β_1_ integrin (23). Indeed, analysis of the fraction of activated integrin (FN7-10 binding) on the surface of these cells in the SCs of EAE mice treated prophylactically with either mIgG1 or Ang2 Ab at the disease peak revealed that Ang2 blockade inhibits α_5_β_1_ integrin activation in microglia, but not in macrophages (Figure 4C). Taken together, these data suggest that Ang2 modulates inflammatory responses of CNS myeloid cells, particularly in microglia, at least in part via α_5_β_1_ integrin in the CNS autoimmune disease.

### Ang2 blockade downregulates vascular inflammation within the CNS.

**Figure 5:**
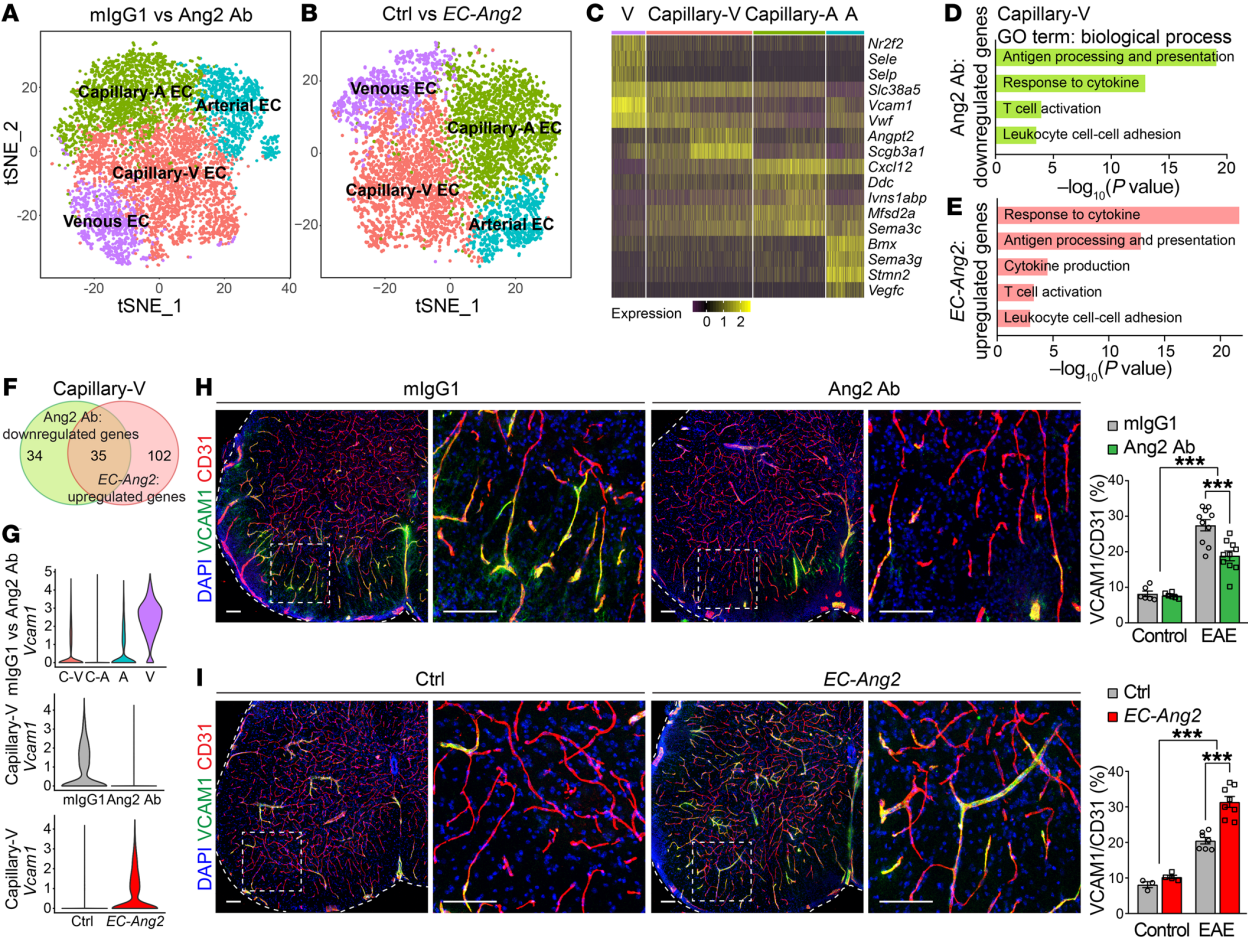
Prophylactic Ang2 blockade suppresses vascular inflammation in the SCs of EAE mice. (**A** and **B**) t-SNE analysis of the main EC clusters (venous and arterial) in the SCs of mIgG1- versus Ang2 Ab–treated EAE mice at 14 dpi and single transgenic control versus *EC-Ang2* EAE mice at 12 dpi. capillary-V, capillary-venous; capillary-A, capillary-arterial. (**C**) Heatmap showing log_2_ expression of known marker genes in each cluster. (**D** and **E**) Relevant GO biological processes of significantly (adjusted *P* < 0.05) downregulated genes after Ang2 blockade (**D**) and upregulated genes after Ang2 overexpression (**E**) in the SC capillary-venous ECs. (**F**) Venn diagram illustrating the number of genes that were regulated by Ang2 in the SC capillary-venous ECs. (**G**) Violin plots showing *Vcam1* expression in different EC clusters as well as its differential expression in capillary-venous ECs regulated by Ang2. (**H** and **I**) Representative images (EAE) and quantification of VCAM1 in the SC blood vessels of mIgG1- versus Ang2 Ab–treated control (*n* = 6 per group) and EAE (*n* = 10 per group) mice at 14 dpi and control versus *EC-Ang2* control (Ctrl, *n* = 3; *EC-Ang2*, *n* = 4) and EAE (Ctrl, *n* = 7; *EC-Ang2*, *n* = 8) mice at 12 dpi. Scale bars: 100 μm. Mean ± SEM, 2-way ANOVA with Bonferroni’s post hoc test for multiple comparisons (**H** and **I**). ***P < 0.001.

It has been reported that EAE induces angiogenesis in the CNS (24). Consistent with the involvement of Ang2 in vascular remodeling in inflammation (25, 26), we found that Ang2 blockade inhibited EAE-induced increase in blood vessel density and diameter in the SC white matter at 14 dpi (Supplemental Figure 3, A and B). However, no differences in SC blood vessels were observed between mIgG1- and Ang2 Ab–treated control mice (Supplemental Figure 3, A–C). To understand the molecular mechanisms underlying the effect of Ang2 on EC-mediated leukocyte infiltration during neuroinflammation, we performed scRNA-Seq analysis of CD31^+^ ECs sorted from the SCs of mIgG1- versus Ang2 Ab–treated EAE mice at 14 dpi and single transgenic control versus *EC-Ang2* EAE mice at 12 dpi (Supplemental Figure 2B). Hierarchical clustering of the cells identified 4 EC clusters (Figure 5, A and B). Based on their distinct gene expression patterns, the EC clusters consisted of venous (*Nr2f2*, *Sele*, *Selp*, *Slc38a5*, *Vcam1*, and *Vwf*), capillary-venous (*Angpt2* and *Scgb3a1*), capillary-arterial (*Cxcl12*, *Ddc*, *Ivns1aBp*, and *Msfd2a*), and arterial (*Bmx*, *Sema3g*, *Stmn2*, and *Vegfc*) ECs (27–30) (Figure 5C). The capillary-venous ECs showed the largest number of significantly (adjusted *P* < 0.05) downregulated genes, followed by capillary-arterial, arterial, and venous ECs by Ang2 blockade. Endogenous *Ang2* was most highly expressed in the capillary-venous EC cluster (Figure 5C). GO biological process analysis of genes that were significantly downregulated in the capillary-venous ECs of Ang2 Ab–treated mice revealed their involvement in leukocyte cell-cell adhesion, antigen processing and presentation, response to cytokine, and T cell activation (Figure 5D). Interestingly, consistent with reports showing MHCII expression in ECs in several autoimmune diseases (31, 32), we also detected EC expression of MHCII genes involved in antigen processing and presentation, which were downregulated by Ang2 blockade in EAE mice.

In contrast to the effect of Ang2 blockade, Ang2 overexpression led to upregulation of the corresponding GO biological processes in the capillary-venous ECs (Figure 5E). Altogether, 35 genes were inversely regulated in the capillary-venous ECs by both Ang2 blockade and overexpression (Figure 5F and Supplemental Table 1). Among these genes, the vascular cell adhesion molecule *Vcam1* was highly expressed in the venous EC cluster, but also expressed in the arterial and capillary-venous EC clusters. *Vcam1* was downregulated in the capillary-venous ECs after Ang2 blockade and upregulated in the Ang2-overexpressing mice (Figure 5G). In addition, Ang2 blockade downregulated *Vcam1* and *Icam2* in arterial ECs, and Ang2 overexpression upregulated *Vcam1* and *Icam1* expression in capillary-arterial and *Selp* in venous EC clusters (Supplemental Figure 4, A and B). RT-qPCR confirmed that Ang2 blockade led to downregulation of *Sele*, *Selp*, *Vcam1*, *Icam1*, and *Pecam1* in the SCs of EAE mice (Supplemental Figure 4C). Furthermore, immunostaining of SCs showed that VCAM1 and P-selectin were more abundant in the SC vasculature of EAE mice than of control mice; both were decreased after Ang2 blockade and increased by Ang2 overexpression (Figure 5, H and I, and Supplemental Figure 4, D and E). However, Ang2 blockade had no effects on the expression of EC adhesion molecules in control mice (Figure 5H and Supplemental Figure 4D).

### Ang2 blockade inhibits neuroinflammation-induced BBB leakage.

**Figure 6:**
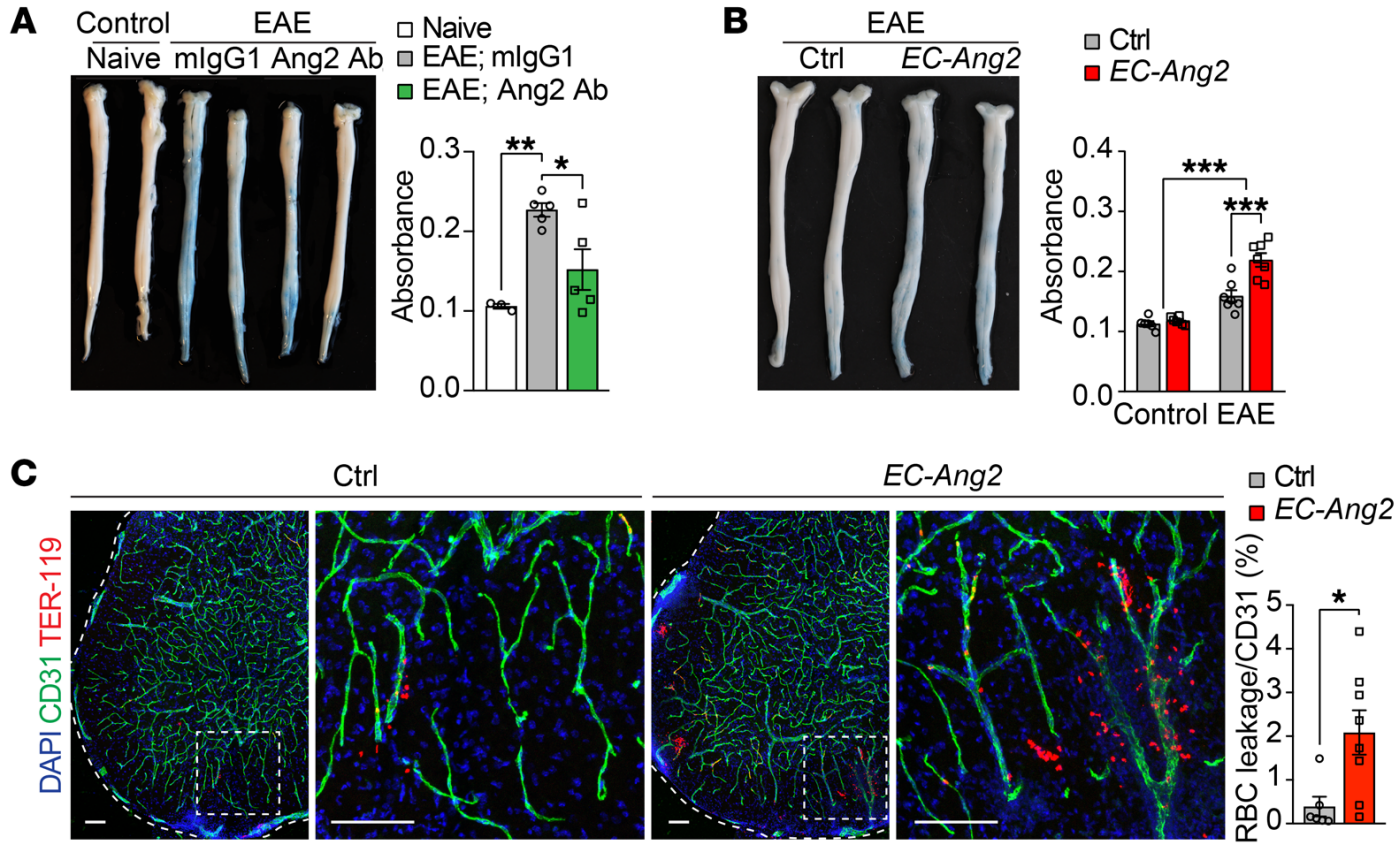
Prophylactic Ang2 blockade improves vascular integrity in the SCs of EAE mice. (**A** and **B**) Representative images and quantifications of Evans blue leakage in the SCs of naive (*n* = 3), mIgG1- versus Ang2 Ab–treated EAE mice (*n* = 5 per group) and control versus *EC-Ang2* control (*n* = 6 per group) and EAE (*n* = 7 per group) mice at 12 dpi. (**C**) Representative images and quantification of extravascular TER-119^+^ RBCs in the SCs of control (*n* = 6) versus *EC-Ang2* (*n* = 8) EAE mice at 12 dpi. Scale bars: 100 μm. Mean ± SEM, 1- or 2-way ANOVA with Bonferroni’s post hoc test for multiple comparisons (**A** and **B**) and 2-tailed Student’s *t* test (**C**). *P < 0.05; **P < 0.01; ***P < 0.001.

To investigate the effect of Ang2 on vascular integrity in the CNS, we analyzed the leakage of intravenously injected Evans blue into the SCs of EAE mice. We found that the neuroinflammation-induced BBB leakage was suppressed in the Ang2 Ab–treated EAE mice (Figure 6A). Ang2 overexpression in ECs did not induce BBB leakage of Evans blue as such, but in the context of neuroinflammation, it led to more severe leakage (Figure 6B). The Ang2-overexpressing EAE mice also had more extravasated TER-119^+^ erythrocytes in the SCs than the single transgenic control mice (Figure 6C), indicating increased BBB permeability upon Ang2 overexpression. Collectively, these data suggest that Ang2 promotes leukocyte recruitment into the CNS via upregulation of EC adhesion molecules and induction of vessel destabilization in neuroinflammation.

### Tie2 activation by Ang2-binding and Tie2-activating Ab ameliorates autoimmune neuroinflammation.

**Figure 7:**
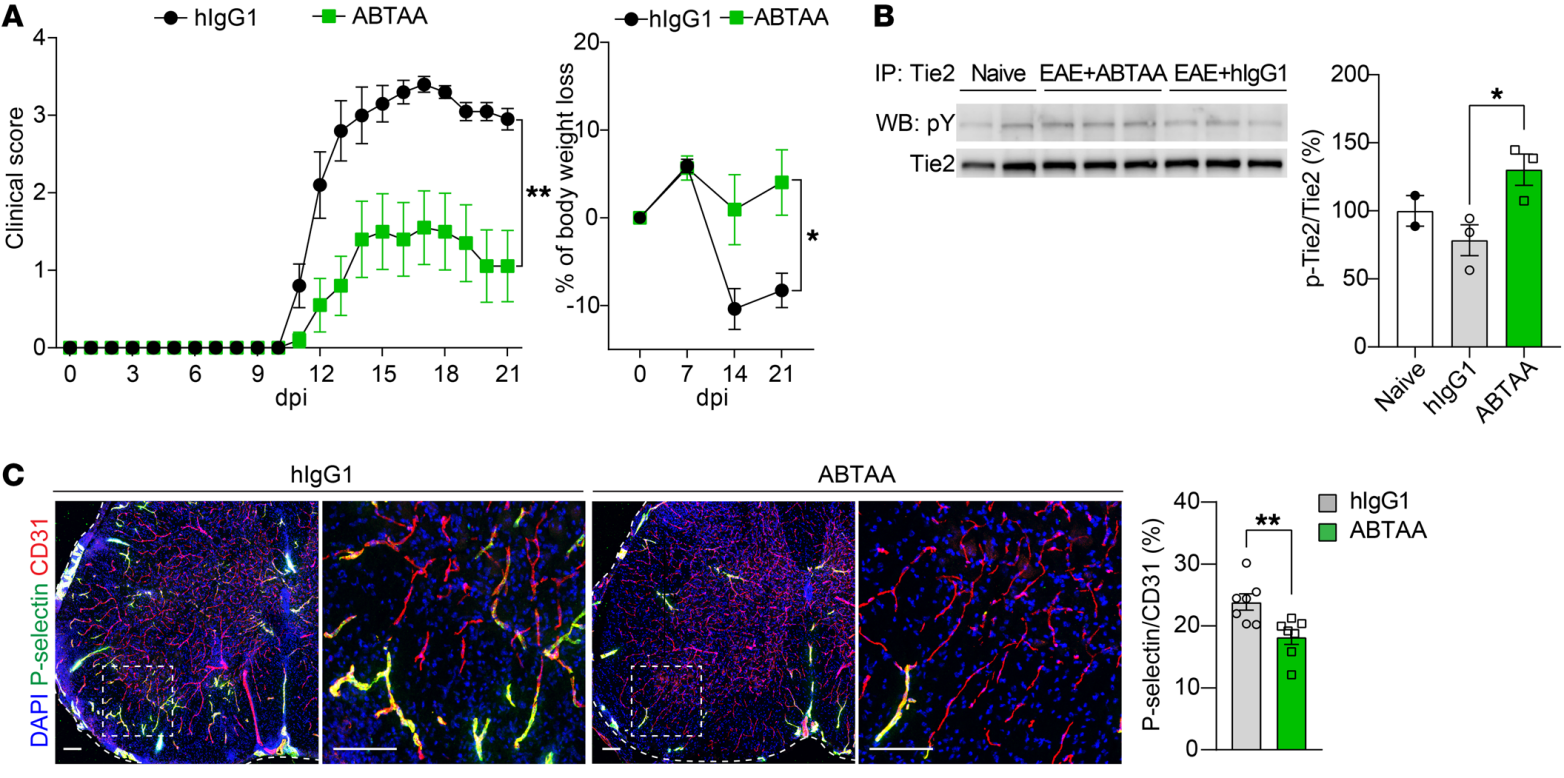
Prophylactic ABTAA induces Tie2 activation and ameliorates EAE. (**A**) Clinical scores and percentages of body weight loss of mice induced with active EAE and treated with hIgG1 versus ABTAA prophylactically (Ab administration started at the time of EAE induction, 0 dpi) (*n* = 10 per group). (**B**) Tie2 phosphorylation in the lungs of EAE mice treated with hIgG1 versus ABTAA prophylactically (*n* = 3 per group) at 14 dpi by immunoprecipitation and Western blot (WB) detections with anti-phosphotyrosine (pY) and anti-Tie2 Abs. (**C**) Representative images and quantification of P-selectin in the SC blood vessels of hIgG1- versus ABTAA-treated EAE mice (*n* = 7 per group) at 14 dpi. Scale bars: 100 μm. Mean ± SEM, nonparametric Mann-Whitney *U* test (**A**, comparison of AUC values of clinical EAE scores over the disease course), 2-way repeated measures ANOVA (**A**, percentage of body weight loss), 1-way ANOVA with Bonferroni’s post hoc test for multiple comparisons (**B**), and 2-tailed Student’s *t* test (**C**). *P < 0.05; **P < 0.01.

It has been previously shown that Ang2 can antagonize Tie2 phosphorylation, thereby inducing vascular instability and leakage (33, 34). Ang2-binding and Tie2-activating Ab (ABTAA) has been reported to be more effective than conventional Ang2-blocking Abs in alleviating sepsis and normalizing tumor vessels (16, 33). To explore whether increasing Tie2 activation alleviates neuroinflammation, we induced active EAE in mice that were treated prophylactically with human IgG1 (hIgG1) isotype control or ABTAA. As shown in Figure 7A, ABTAA ameliorated the clinical severity of disease and attenuated the body weight loss in comparison with that of hIgG1-treated control mice. Analysis of Tie2 phosphorylation in the lungs of hIgG1- versus ABTAA-treated EAE mice at 14 dpi showed that ABTAA promoted Tie2 activation in neuroinflammation (Figure 7B). Furthermore, immunostaining revealed that ABTAA downregulated P-selectin in the SC blood vessels of these mice (Figure 7C). Taken together, these data indicate that converting Ang2 antagonization to Tie2 activation ameliorates autoimmune neuroinflammation.

## Discussion

In this study, we identified a previously unrecognized role of Ang2 in autoimmune neuroinflammation. Our results show that Ang2, a major regulator of vascular permeability, has an essential role in the pathogenesis of CNS autoimmune disease. Ang2 expression was induced in neuroinflammation and transgenic mice overexpressing an Ang2 transgene specifically in ECs exacerbated EAE. In contrast, administration of the Ang2-blocking Ab ameliorated the progression of neuroinflammation and decreased SC demyelination and leukocyte infiltration into the CNS. Moreover, Ang2 blockade inhibited expression of EC adhesion molecules, improved BBB integrity, and decreased proinflammatory responses in CNS myeloid cells, which was accompanied by inhibition of α_5_β_1_ integrin activation in microglia.

The increased concentration of Ang2 in the serum and CNS of EAE mice is consistent with previous studies showing that inflammatory signals induce increased Ang2 expression and release in ECs (35, 36). Elevated systemic Ang2 is known to promote vascular leakage and to lead to amplification of the effects of other proinflammatory cytokines (4, 13). Increased Ang2 serum levels in patients have been shown to correlate with poor prognosis in sepsis, diabetic retinopathy, and cancer (4). Although 2 recent studies reported elevated Ang2 concentration in the brain and serum of MS patients (37, 38), no studies have previously addressed the function of Ang2 in CNS autoimmune diseases.

A major neuroprotective mechanism associated with the amelioration of autoimmune neuroinflammation by Ang2 blockade was inhibition of leukocyte infiltration into the CNS as analyzed by flow cytometry, immunohistochemistry, and intravital 2-photon laser scanning microscopy. Interestingly, although Ang2 blockade attenuated T cell infiltration in neuroinflammation, in the setting of tumor angiogenesis, dual Ang2 and VEGFA inhibition has been shown to promote vascular normalization, thereby facilitating recruitment of T cells into the tumors (18, 39). Microglia and macrophages are the most important CNS myeloid cells in the pathogenesis of EAE and MS (40–43). According to current views, infiltrating autoimmune T cells and tissue-resident microglia are responsible for EAE initiation, and infiltrating macrophages contribute to EAE and MS progression (44). Our scRNA-Seq analysis showed that Ang2 blockade leads to downregulation of genes involved in antigen presentation and proinflammatory responses in both microglia and macrophages. Moreover, Ang2 blockade downregulated the expression of genes promoting APOE-induced microglial and macrophage neurodegenerative phenotype, but not the expression of homeostatic genes or transcription factors in these cells (21). In addition to the effect of Ang2 blockade in dampening proinflammatory processes in myeloid cells, it also downregulated the expression of genes induced by oxidative stress that are associated with neurotoxicity (20). This indicates that Ang2 blockade restores a neuroprotective microenvironment in the inflamed CNS.

In agreement with our results, previous studies have shown that Ang2 is expressed by macrophages (11, 45) and that it promotes macrophage proinflammatory polarization via α_5_β_1_ integrin, thereby exacerbating inflammation after myocardial ischemia (11). Ang2 may also act directly on macrophages and neutrophils in a paracrine manner (46–48). According to our results, the CNS myeloid cells expressed much lower levels of Ang2 than ECs. Furthermore, Ang2 overexpression in ECs exacerbated EAE, whereas both Ang2-blocking Ab and ABTAA, which induces Tie2 activation in ECs (16, 49), ameliorated EAE. These data suggest that the EC-derived Ang2 is mainly responsible for the aggravation of neuroinflammation. However, the exact contribution of Ang2 derived from ECs versus CNS myeloid cells in neuroinflammation remains to be determined. Microglia and macrophages expressed much lower levels of Tie2 than ECs, but all 3 cell types expressed comparable levels of α_5_β_1_ integrin, the activation of which was inhibited by the Ang2-blocking Ab. Thus, the beneficial effects of Ang2 blockade in neuroinflammation may be partly mediated by its antiinflammatory effects targeting α_5_β_1_ integrin in CNS myeloid cells. Future studies using dual reporter mice or bone marrow chimeras that allow better distinction of microglia from macrophages should provide further insights into Ang2 functions in the regulation of myeloid cells in neuroinflammation (50, 51).

Elevated levels of adhesion molecules in the CNS are correlated with the severity of EAE and MS (52). In our study, Ang2 blockade downregulated neuroinflammation-induced EC adhesion molecules, leading to decreased leukocyte infiltration into the CNS. In contrast, Ang2 overexpression upregulated EC adhesion molecules and led to exacerbated leukocyte infiltration into the SCs. In addition to EC adhesion molecules, BBB permeability also modulates leukocyte influx into the CNS parenchyma (2). BBB is a highly selective barrier comprising nonfenestrated ECs interconnected with tight junctions, pericytes, and astrocyte endfeet, which makes it unique in comparison with the peripheral vascular system (1). In EAE, compromised BBB promotes initial infiltration of autoimmune T cells, which are reactivated when they encounter local antigen-presenting cells in the CNS parenchyma, leading to an inflammatory cascade that results in axonal and myelin damage (3). Disruption of BBB also leads to extravasation of fibrinogen into the CNS parenchyma, where it is converted to fibrin that activates innate immunity (53). A monoclonal Ab against fibrin has been shown to attenuate inflammation and oxidative stress in neuroinflammatory and neurodegenerative diseases (54). In myocardial infarction and sepsis mouse models, Ang2 has been shown to activate α_5_β_1_ integrin and to antagonize Tie2, resulting in abnormal vascular remodeling and vascular leakage (11, 14, 22). In contrast, Ang1 treatment inhibited vascular leakage and ameliorated EAE disease development (55). Consistent with these findings, our experiments showed that multimerization of Ang2 by ABTAA increased Tie2 activation and ameliorated CNS autoimmunity.

In summary, our findings demonstrate that Ang2 is critically involved in the CNS autoimmune process. Ang2 blockade ameliorated CNS autoinflammation by downregulating the neuroinflammation-induced upregulation of EC adhesion molecules and improving BBB integrity, thereby attenuating leukocyte recruitment into the CNS. In addition, Ang2 blockade inhibited α_5_β_1_ integrin activation in microglia and the proinflammatory polarization of CNS myeloid cells. Thus, intervention by Ang2 blockade may provide an alternative therapeutic option for the treatment of CNS autoimmune diseases.

## Methods

### Mice.

Nine-week-old C57BL/6JOlaHsd female mice were purchased from Envigo. The *VE-cadherin-tTA* (56) and *Tet-OS-Ang2* (17) mice (both on C57BL/6J 000664 background) were described previously. *VE-cadherin-tTA* mice were mated with *Tet-OS-Ang2* mice to generate double-transgenic EC-specific Ang2-overexpressing (*EC-Ang2*) mice. Expression of Ang2 transgene was repressed until birth by giving doxycycline-supplemented food (ssniff Spezialdiäten GmbH) to pregnant females. Single-transgenic littermates were used as controls. Animals were kept under standard breeding conditions with food and water given ad libitum and acclimated to the local animal facility for at least 1 week before the experiments.

### Ab administration.

mIgG1 isotype control Ab (Eli Lilly and Co.), mouse anti-mouse Ang2 Ab (18E5, Eli Lilly and Co.), hIgG1 isotype control Ab (Synagis, AbbVie), and ABTAA (16) were administrated i.p. at a dose of 20–25 mg/kg body weight 2 to 3 times a week.

### Active EAE.

Approximately 10- to 12-week-old C57BL/6J mice were immunized with subcutaneous injections of an emulsion of 200 μg of MOG_35-55_ peptide in CFA into 2 sites on the back. This was followed by i.p. administration of 2 doses of 400 ng of lyophilized pertussis toxin (200 ng for single transgenic control and *EC-Ang2* mice) dissolved in PBS on the same and the following day after immunization with the EAE induction kit (Hooke Laboratories, EK-2110) according to the manufacturer’s instructions. Starting from 7 dpi, mice were monitored daily by 2 investigators for clinical signs of paralysis. The scoring criteria used for active EAE were as follows: 0, no clinical symptoms; 0.5, partially limp tail; 1, limp tail; 1.5, limp tail and 1 hind leg paresis; 2, limp tail and weakness of hind legs; 2.5, limp tail and dragging of hind legs; 3, limp tail and complete paralysis of hind legs; 3.5, limp tail, complete paralysis of hind legs, and weakness of front legs; and 4, complete paralysis of both hind and front legs (57).

### Adoptive transfer EAE.

Adoptive transfer EAE was performed as previously described (58). Briefly, donor GFP C57BL6/J mice were immunized with 75 μg of MOG_35--55_ peptide in CFA, and 200 ng of Bordetella pertussis toxin was administered i.p. at 0 and 2 dpi. Draining lymph nodes were collected at 12 dpi, a single-cell suspension was prepared, and the cells were cultivated for 3 days in the presence of 25 μg/mL MOG_35–55_ peptide, 25 ng/mL recombinant mouse IL-12 (R&D Systems), and 20 μg/mL α-IFN-γ Ab (clone XMG1.2, BioXCell). After 3 days, cells were collected, counted and transferred into naive C57BL6/J recipient mice (2.5 × 10^6^ cells/mouse). The scoring criteria used for adoptive transfer EAE were as follows: 0, healthy; 1, reduced tail tone, paralysis of the tip of the tail; 2, flaccid tail paralysis; 3, loss of righting reflex; 4, gait ataxia; 5, mild paralysis of the hind limbs; 6, moderate paralysis of hind limbs or full paralysis of only 1 hind leg; 7, paralysis of both hind limbs; 8, tetraparesis; 9, moribund; and 10, dead (58).

### In vivo 2-photon imaging of mouse SC.

In vivo 2-photon imaging was performed as previously described (58, 59). Briefly, animals were anesthetized by 1 mg/kg medetomidine and 100 mg/kg ketamine, tracheally intubated, ventilated, and stabilized in a custom-made heated microscope stage. Body temperature was monitored with a rectal thermometer (Telemeter Electronic GmbH) and was kept constant at 36–37°C. The lower thoracic SC was accessed by performing a laminectomy at level Th12/L1 as previously described (59). The animals were imaged at the onset and peak of disease, i.e., between day 6 and day 13 after transfer. Blood vessels were visualized by i.v. injection of fluorescently labeled 2000 kDa dextran. Intravital 2-photon recordings were performed with an LSM710/Axio Examiner Z1 microscope (Carl Zeiss) and a greater than 2.5-watt Ti:Sapphire Chameleon Vision II Laser (Coherent GmbH). Stacks were acquired with 4 μm intervals apart on the *z* axis and scan size of 512 × 512 pixels. Each stack was acquired for 58 cycles with a 32-second time interval and 2 line averaging. Four-dimensional stacks were analyzed with Imaris software (Bitplane). Motility parameters were exported into Microsoft Excel and plotted using GraphPad Prism 8.0.

### Assessment of vascular integrity by analysis of Evans blue extravasation.

At 12 dpi, mice were i.v. injected with 100 μL of 3% Evans blue in PBS. Three hours later, mice were anesthetized and perfused with ice-cold PBS; SCs were then dissected. The tissue was then cut into small pieces with scalpels, and Evans blue was extracted by incubating the tissue in deionized formamide at 56°C overnight. After centrifugation at 16,100 *g* at room temperature (RT) for 15 minutes, supernatant from the homogenate was carefully taken, filtered through a 70-μm cell strainer (Fisher Scientific), and transferred to a fresh 1.5-mL Eppendorf tube. The optical densities of formamide extracts from SC tissues at 620 nm and 740 nm were measured on an EnSight Multimode Plate Reader (PerkinElmer). Evans blue absorbance was corrected for turbidity by subtracting the optical density at 740 nm from the total optical density at 620 nm (60).

### ELISA.

The SCs were snap-frozen in liquid nitrogen and stored at –80°C until use. After homogenizing the tissue with R&D Sample Diluent (0.5% NP-40 Alternative, 10 mM Tris-HCl [pH 8.0], 68.5 mM NaCl, 5% glycerol, 1 mM EDTA, 0.5 mM activated Na_3_VO_4_) in a 2-mL screw-cap tube containing zirconium oxide beads (MB2Z015, Biotop) on a PowerLyzer 24 homogenizer (MO BIO Laboratories), supernatant was collected by centrifugation at 16,100 *g* at 4°C for 15 minutes. Ang2 concentration in the serum and SC lysates was measured by Quantikine ELISA Mouse/Rat Ang2 Immunoassay Kit (R&D Systems) according to the manufacturer’s instructions.

### Flow cytometry.

At indicated time points after EAE induction, mice were anesthetized with ketamine and xylazine and perfused transcardially with ice-cold PBS; SCs were then dissected. To analyze leukocyte infiltration into the CNS, the tissues were cut into tiny pieces and mechanically dissociated with the plunger of a 3-mL syringe through 70-μm cell strainers (Fisher Scientific). Mononuclear cells were enriched by discontinuous Percoll (GE Healthcare) gradient centrifugation, as described previously (19). To compare Tie2 and α_5_ integrin expression, ECs and immune cells were enriched by using the Neural Tissue Dissociation Kit (P) and Myelin Removal Beads II (Miltenyi Biotec), as described previously (27). Cells were first blocked with Mouse BD Fc Block (clone 2.4G2, BD Pharmingen) on ice for 5 minutes and then stained on ice for another 30 minutes with a combination of the following fluorophore-conjugated anti-mouse Abs (all Abs were purchased from BioLegend unless otherwise indicated): CD45-FITC (clone 30-F11), CD3-PE (clone 145-2C11), CD202b (Tie2)-PE (clone TEK4), CD8-PerCP/Cy5.5 (clone 53-6.7), CD4-PE/Cy7 (clone GK1.5), CD49e (α_5_ integrin)-PE/Cy7 (clone 5H10-27 [MFR5]), CD31-APC (clone MEC13.3, BD), MHCII-APC (clone M5/114.15.2), MHCII–Alexa Fluor 700 (clone M5/114.15.2), Ly-6C–Alexa Fluor 700 (clone HK1.4), Ly-6G-APC/Cy7 (clone 1A8), F4/80-Brilliant Violet 421 (clone BM8), and CD11b-Brilliant Violet 510 (clone M1/70). After washing with FACS buffer (PBS supplemented with 1% FBS, 1 mM EDTA, and 0.05% NaN_3_), stained cells were resuspended in FACS buffer and acquired on a FACSAria II flow cytometer (BD Biosciences). Flow cytometric data were analyzed with Kaluza Analysis Software (Beckman Coulter). The number of immune cells is presented as number of cells per SC. The surface expression levels of Tie2 and α_5_ integrin are presented as geometric mean fluorescence intensity (GMFI).

### Integrin activation assay.

Flow cytometry analyzing the binding of cells to fluorescently labeled FN7-10 was performed to assess active levels of α_5_β_1_ integrin on the cell surface (61, 62). Briefly, at indicated time points after EAE induction, mice were anesthetized with ketamine and xylazine and perfused transcardially with ice-cold PBS; SCs were then dissected. The tissue was cut into tiny pieces and mechanically dissociated with the plunger of a 3-mL syringe through 70-μm cell strainers (Fisher Scientific). Mononuclear cells were enriched using discontinuous Percoll gradients. Cells were resuspended in RPMI 1640 (Corning) and blocked with Mouse BD Fc Block at RT for 5 minutes. Each sample was subsequently stained with (a) 2% Alexa Fluor 647–conjugated FN7-10 (a gift from Pipsa Saharinen, University of Helsinki, Helsinki, Finland), (b) 2% Alexa Fluor 647–conjugated FN7-10 supplemented with 10 mM EDTA, or (c) 1% CD49e-PE/Cy7 (recognizing total α_5_β_1_) in combination with the following fluorophore-conjugated anti-mouse Abs: CD45-FITC, Ly-6C-Alexa Fluor 700, Ly-6G-APC/Cy7, F4/80-Brilliant Violet 421, and CD11b-Brilliant Violet 510 at RT with slow agitation for another 30 minutes. After washing once with cold Tyrode’s buffer (10 mM HEPES buffer, 137 mM NaCl, 2.68 mM KCl, 0.42 mM NaH_2_PO_4_, 1.7 mM MgCl_2_, 11.9 mM NaHCO_3_, 5 mM glucose, 0.1% BSA, pH 7.5), cells were resuspended in cold Tyrode’s buffer and analyzed on a FACSAria II flow cytometer. The α_5_β_1_ integrin activation index was calculated by measuring GMFI of activated β_1_ integrin (Alexa Fluor 647–conjugated FN7-10 binding) subtracted from a background signal (Alexa Fluor 647–conjugated FN7-10 binding in the presence of inactivating EDTA) relative to total cellular α_5_ integrin in the respective cells.

### Total RNA isolation and real-time RT-qPCR.

SCs were snap-frozen in liquid nitrogen and stored at –80°C until further use. After homogenizing the tissue with TRIsure (Bioline) in a 2-mL screw-cap tube containing zirconium oxide beads on a PowerLyzer 24 homogenizer, chloroform was added and the homogenate was separated into 3 phases by centrifugation at 12,000 *g* at 4°C for 5 minutes. Total RNA was extracted from the upper aqueous phase using a NucleoSpin RNA Isolation Kit (Macherey-Nagel) according to the manufacturer’s instructions, with modifications. The quality (A260/A280 > 2.0) and concentration of total RNA samples was determined by a Nanodrop ND-1000 spectrophotometer. Total RNA was reverse-transcribed into cDNA with the High Capacity cDNA Reverse Transcription Kit (Applied Biosystems). RT-qPCR was performed with corresponding primer pairs and FastStart Universal SYBR Green Master Mix (Roche) on the CFX384 Real-Time PCR Detection System (Bio-Rad). The forward and reverse primers used are listed in Supplemental Table 2. Relative mRNA expression levels (2^-ΔΔCt^) of each gene were calculated against *Rplp0* or *Hprt* and then set as fold change against its expression in the SCs of mIgG1-treated EAE mice.

### scRNA-Seq of immune cells and ECs from SC of EAE mice.

CD45^+^ immune cells and CD31^+^ ECs were enriched from the SCs by using the Neural Tissue Dissociation Kit (P) and Myelin Removal Beads II (Miltenyi Biotec) as described previously (27). Briefly, after enzymatic digestion, mechanical dissociation, and myelin debris removal, eluted cells were incubated with Mouse BD Fc Block for 5 minutes and then stained with CD45-FITC and CD31-APC on ice for 30 minutes. DAPI was added to the cell suspension right before sorting. Equal numbers of viable DAPI^–^CD45^+^ immune cells and DAPI^–^CD31^+^ ECs were sorted with BD Influx Cell Sorter (BD Biosciences) into PBS supplemented with 0.04% BSA. Cell concentration and viability were determined in a Countess II Automated Cell Counter with LIVE/DEAD Viability/Cytotoxicity Kit (Life Technologies). Approximately 10,000 sorted cells were further processed for scRNA-Seq using the Chromium Single Cell 3′ Reagent Kit, version 2, and the Gel Bead Kit, version 2 (10× Genomics), according to the manufacturer’s instructions. Libraries were sequenced to an average depth of 50,000 reads on a Novaseq 6000 Sequencing System (Illumina), and raw sequencing data were processed with Cell Ranger analysis pipelines.

ScRNA-Seq data were analyzed with the Seurat package 2.3 in R (63). After filtering out cellular doublets, high mitochondrial gene-containing cells, and cells undergoing cell cycle in each sample, approximately 2000 highly variable genes between the 2 samples were identified and selected for further analysis. The R objects were then merged, and dimensions were reduced with the canonical correlation analysis (CCA). The subspace among the cells was aligned, and cell clusters were identified and visualized with a t-SNE plot based on a graph-based clustering approach. scRNA-Seq data for *Ptprc*^+^ immune cell clusters and *Pecam1*^+^ EC clusters were subsetted, the subspace among these cells was realigned, and cell clusters were reidentified and visualized in a t-SNE plot. Contaminating pericytes and cells that formed extremely small clusters were removed by supervised gating of the t-SNE plot. Colors illustrate unbiased immune cell or EC classification. Conserved markers were identified with the FindConservedMarkers function, and differentially expressed genes between samples in each cluster were identified with the FindMarkers function in Seurat (63).

### GO biological process analysis.

The GO term biological processes of the significantly differentially expressed genes (adjusted *P* < 0.05) were analyzed with the functional annotation clustering tool in the Database for Annotation, Visualization and Integrated Discovery (DAVID) (64, 65).

### Immunohistochemical staining of SC sections.

For preparation of SCs for immunohistochemical staining, mice were given a lethal dose of ketamine and xylazine and transcardially perfused with ice-cold PBS or 1% PFA in PBS. SCs were carefully dissected and embedded in OCT Cryomount (Histolab) in cryomolds (Sakura) that were frozen in a bath of 2% pentane in methylbutane (Sigma-Aldrich), cooled on dry ice, and stored at –80°C until further use. Alternatively, SCs were dissected and fixed with 4% PFA in PBS at 4°C overnight. After 30% sucrose dehydration, SCs were embedded in OCT Cryomount in cryomolds, frozen on dry ice, and stored at –80°C until further use. SCs were cut into 20- or 50-μm cryosections in a cryostat, mounted onto Superfrost Plus glass slides (Thermo Scientific), and stored at –20°C. For immunofluorescence staining, sections were air-dried at RT for 30 minutes, fixed with 1% PFA in PBS for 15 minutes, and permeabilized in PBS containing 0.3% Triton X-100. Following blocking with donkey immunomix (DIM) (5% donkey serum, 0.2% BSA, 0.3% Triton X-100, 0.05% NaN_3_ in PBS) at RT for 1 hour, sections were incubated with primary Abs in DIM at 4°C for 1 to 2 nights. The following primary Abs were used: rat anti-MBP (clone 12, Bio-Rad), rabbit anti-Iba1 (Wako, catalog 019-19741), goat anti-Ly-6G (clone 1A8, BioLegend), rat anti-CD4 (clone RM4-5, BD Pharmingen), rat anti-I-A/I-E (clone M5/114.15.2, BioLegend), goat anti-CD31 (R&D Systems, catalog AF3628), rat anti-VCAM1 (clone M/K-2, Merck), rat anti-CD31 (clone MEC 13.3, BD Pharmingen), rat anti-mouse TER-119 (clone TER-119, BD Pharmingen), and goat anti-P-selectin (R&D Systems, catalog AF737). After washing with PBS containing 0.1–0.3% Triton X-100, sections were stained with fluorophore-conjugated secondary Abs in PBS containing 0.1% Triton X-100 or in DIM at RT. Following DAPI staining, sections were washed with PBS containing 0.1% Triton X-100 and PBS. The sections were post-fixed with 1% PFA in PBS, mounted with VectaShield (Vecta Laboratories), and sealed with Cytoseal (Thermo Scientific).

### Image acquisition and quantification.

Immunofluorescent images of SC sections were acquired with an Axio Imager (Carl Zeiss) through Hamamatsu Orca Flash 4.0 LT camera and Zen Pro 2.3 software. Laser scanning confocal *Z*-stack images of the fluorescently labeled SC cryosections were acquired with Zen 2012 software (Carl Zeiss) using a Carl Zeiss LSM 780 or 880 confocal microscope (air objectives 10× Plan-Apochromat with NA 0.45 and 20× Plan-Apochromat with NA 0.80) with multichannel scanning in tiles. The *Z*-stack images were rendered to maximum intensity projections or extended depth of focus. Image brightness and contrast were adjusted using Fiji ImageJ (version 1.52b, NIH). Quantification was performed using Fiji ImageJ software and reported as area fraction of region of interest. Vessel density and diameter (total vessel area divided by total vessel length) were quantified using AngioTool (66). EC adhesion molecule expression was calculated as percentage of VCAM1^+^ and P-selectin^+^ area within the CD31^+^ EC area. MHCII expression was calculated as percentage of MHCII^+^ area within the Iba1^+^ area. Extravasated TER-119^+^ red blood cells were calculated as percentage of TER-119^+^ area outside the blood vessels divided by CD31^+^ area.

### Analysis of Tie2 phosphorylation by immunoprecipitation and Western blot.

To analyze Tie2 phosphorylation in the lungs of mice that were induced with EAE and treated prophylactically with Abs, snap-frozen tissues were homogenized with ice-cold RIPA buffer containing protease and phosphatase inhibitors (25 mM Tris-HCl [pH 7.4], 150 mM NaCl, 0.5% Nonidet P-40, 0.5% Triton X-100, 2 mM EDTA, 10.2 μg/mL Aprotinin, 10 μg/mL leupeptin, 1 mM PMSF, 5 mM NaF, and 1 mM Na_3_VO_4_) in a 2-mL screw-cap tube containing zirconium oxide beads on a PowerLyzer 24 homogenizer. Supernatant was collected by centrifugation at 13,400 *g* at 4°C for 15 minutes, and protein concentration was determined using a Pierce BCA Protein Assay Kit (Thermo Fisher). Prewashed Protein G Sepharose 4 Fast Flow Beads (GE Healthcare) were incubated with goat anti-mouse Tie2 Ab (catalog AF762, R&D Systems) at 4°C for 1 hour. The Ab–protein G sepharose beads were then added to the supernatants containing approximately 2.5 mg of total protein at 4°C for 2 hours. After washing, the immunoprecipitates were then boiled in 2× Laemmli buffer, subjected to SDS-PAGE on 8% Tris-Glycine gel, and transferred to nitrocellulose membrane. Following blocking with 5% BSA in PBS, the membrane was first incubated with mouse anti-phosphotyrosine Ab (clone 4G10, Millipore), then with HRP-conjugated secondary Ab, and was developed with SuperSignal West Pico PLUS Chemiluminescent Substrate (Thermo Scientific). The membrane was stripped and reprobed with goat anti-mouse Tie2 Ab.

### Data availability.

All scRNA-Seq data sets were deposited in the NCBI’s Gene Expression Omnibus database (GEO GSE129105).

### Statistics.

Data are expressed as mean ± SEM. Comparisons between 2 groups were analyzed using 2-tailed Student’s *t* test for parametric data. Comparisons among 3 or more groups were analyzed using 1-way ANOVA with Dunnett’s or Bonferroni’s post hoc test for multiple comparisons for parametric data. Comparisons of AUC of clinical EAE scores (nonparametric data) between 2 groups were analyzed using Mann-Whitney *U* test. Comparisons of percentages of body weight loss between 2 groups were analyzed using 2-way repeated measures ANOVA. Comparison of 2 factors was analyzed using 2-way ANOVA with Bonferroni’s post hoc test for multiple comparisons for parametric data. Graphpad Prism 8.0 was used for statistical analysis. Differences were considered significant at *P* < 0.05.

### Study approval.

All animal studies were approved by the Committee for Animal Experiments of the District of Southern Finland, Finland, and the Lower Saxony State Office for Consumer Protection and Food Safety (LAVES), Germany.

## Author contributions

ZL, EAK, BE, AF, and KA designed research. ZL, EAK, AM, JS, EW, HN, SA, and UD performed research. ZL, EAK, AM, and JS analyzed data. SC and GYK provided reagents. JP provided help on scRNA-Seq data analysis. ZL, EAK, and KA wrote the manuscript.

## Supplementary Material

Supplemental data

## Version 1 03/09/2020

Electronic publication

## Version 2 04/01/2020

Print issue publication

## References

[1] Abbott NJ, Patabendige AA, Dolman DE, Yusof SR, Begley DJ (2010). Structure and function of the blood-brain barrier. Neurobiol Dis.

[2] Larochelle C, Alvarez JI, Prat A (2011). How do immune cells overcome the blood-brain barrier in multiple sclerosis?. FEBS Lett.

[3] Constantinescu CS, Farooqi N, O’Brien K, Gran B (2011). Experimental autoimmune encephalomyelitis (EAE) as a model for multiple sclerosis (MS). Br J Pharmacol.

[4] Saharinen P, Eklund L, Alitalo K (2017). Therapeutic targeting of the angiopoietin-TIE pathway. Nat Rev Drug Discov.

[5] Parikh SM (2017). Angiopoietins and Tie2 in vascular inflammation. Curr Opin Hematol.

[6] Yuan HT, Khankin EV, Karumanchi SA, Parikh SM (2009). Angiopoietin 2 is a partial agonist/antagonist of Tie2 signaling in the endothelium. Mol Cell Biol.

[7] Korhonen EA (2016). Tie1 controls angiopoietin function in vascular remodeling and inflammation. J Clin Invest.

[8] Augustin HG, Koh GY, Thurston G, Alitalo K (2009). Control of vascular morphogenesis and homeostasis through the angiopoietin-Tie system. Nat Rev Mol Cell Biol.

[9] Thurston G (2000). Angiopoietin-1 protects the adult vasculature against plasma leakage. Nat Med.

[10] Gurnik S (2016). Angiopoietin-2-induced blood-brain barrier compromise and increased stroke size are rescued by VE-PTP-dependent restoration of Tie2 signaling. Acta Neuropathol.

[11] Lee SJ (2018). Angiopoietin-2 exacerbates cardiac hypoxia and inflammation after myocardial infarction. J Clin Invest.

[12] Ghosh CC (2015). Drug repurposing screen identifies Foxo1-dependent angiopoietin-2 regulation in sepsis. Crit Care Med.

[13] Fiedler U (2006). Angiopoietin-2 sensitizes endothelial cells to TNF-alpha and has a crucial role in the induction of inflammation. Nat Med.

[14] Hakanpaa L (2018). Targeting β1-integrin inhibits vascular leakage in endotoxemia. Proc Natl Acad Sci U S A.

[15] Benest AV (2013). Angiopoietin-2 is critical for cytokine-induced vascular leakage. PLoS One.

[16] Han S (2016). Amelioration of sepsis by TIE2 activation-induced vascular protection. Sci Transl Med.

[17] Holopainen T (2012). Effects of angiopoietin-2-blocking antibody on endothelial cell-cell junctions and lung metastasis. J Natl Cancer Inst.

[18] Schmittnaegel M (2017). Dual angiopoietin-2 and VEGFA inhibition elicits antitumor immunity that is enhanced by PD-1 checkpoint blockade. Sci Transl Med.

[19] Li Z, Ma L, Kulesskaya N, Võikar V, Tian L (2014). Microglia are polarized to M1 type in high-anxiety inbred mice in response to lipopolysaccharide challenge. Brain Behav Immun.

[20] Nathan C, Cunningham-Bussel A (2013). Beyond oxidative stress: an immunologist’s guide to reactive oxygen species. Nat Rev Immunol.

[21] Krasemann S (2017). The TREM2-APOE pathway drives the transcriptional phenotype of dysfunctional microglia in neurodegenerative diseases. Immunity.

[22] Hakanpaa L (2015). Endothelial destabilization by angiopoietin-2 via integrin β1 activation. Nat Commun.

[23] Nishizaka T, Shi Q, Sheetz MP (2000). Position-dependent linkages of fibronectin- integrin-cytoskeleton. Proc Natl Acad Sci U S A.

[24] Kirk SL, Karlik SJ (2003). VEGF and vascular changes in chronic neuroinflammation. J Autoimmun.

[25] Tabruyn SP (2010). Angiopoietin-2-driven vascular remodeling in airway inflammation. Am J Pathol.

[26] Fuxe J (2010). Angiopoietin/Tie2 signaling transforms capillaries into venules primed for leukocyte trafficking in airway inflammation. Am J Pathol.

[27] Vanlandewijck M (2018). A molecular atlas of cell types and zonation in the brain vasculature. Nature.

[28] Sabbagh MF (2018). Transcriptional and epigenomic landscapes of CNS and non-CNS vascular endothelial cells. Elife.

[29] Sorriento D, Santulli G, Del Giudice C, Anastasio A, Trimarco B, Iaccarino G (2012). Endothelial cells are able to synthesize and release catecholamines both in vitro and in vivo. Hypertension.

[30] Cavallero S, Shen H, Yi C, Lien CL, Kumar SR, Sucov HM (2015). CXCL12 signaling is essential for maturation of the ventricular coronary endothelial plexus and establishment of functional coronary circulation. Dev Cell.

[31] Turesson C (2004). Endothelial expression of MHC class II molecules in autoimmune disease. Curr Pharm Des.

[32] Pober JS, Merola J, Liu R, Manes TD (2017). Antigen presentation by vascular cells. Front Immunol.

[33] Park JS (2016). Normalization of tumor vessels by Tie2 activation and Ang2 inhibition enhances drug delivery and produces a favorable tumor microenvironment. Cancer Cell.

[34] Fiedler U, Augustin HG (2006). Angiopoietins: a link between angiogenesis and inflammation. Trends Immunol.

[35] Kim M (2016). Opposing actions of angiopoietin-2 on Tie2 signaling and FOXO1 activation. J Clin Invest.

[36] Fiedler U (2004). The Tie-2 ligand angiopoietin-2 is stored in and rapidly released upon stimulation from endothelial cell Weibel-Palade bodies. Blood.

[37] Karampoor S, Zahednasab H, Ramagopalan S, Mehrpour M, Keyvani H (2016). Angiogenic factors are associated with multiple sclerosis. J Neuroimmunol.

[38] Chaitanya GV (2013). Inflammation induces neuro-lymphatic protein expression in multiple sclerosis brain neurovasculature. J Neuroinflammation.

[39] De Palma M, Jain RK (2017). CD4^+^ T cell activation and vascular normalization: two sides of the same coin?. Immunity.

[40] Raivich G, Banati R (2004). Brain microglia and blood-derived macrophages: molecular profiles and functional roles in multiple sclerosis and animal models of autoimmune demyelinating disease. Brain Res Brain Res Rev.

[41] Brück W (1995). Monocyte/macrophage differentiation in early multiple sclerosis lesions. Ann Neurol.

[42] Li H, Cuzner ML, Newcombe J (1996). Microglia-derived macrophages in early multiple sclerosis plaques. Neuropathol Appl Neurobiol.

[43] McCombe PA, de Jersey J, Pender MP (1994). Inflammatory cells, microglia and MHC class II antigen-positive cells in the spinal cord of Lewis rats with acute and chronic relapsing experimental autoimmune encephalomyelitis. J Neuroimmunol.

[44] Ajami B, Bennett JL, Krieger C, McNagny KM, Rossi FM (2011). Infiltrating monocytes trigger EAE progression, but do not contribute to the resident microglia pool. Nat Neurosci.

[45] Fang HY (2009). Hypoxia-inducible factors 1 and 2 are important transcriptional effectors in primary macrophages experiencing hypoxia. Blood.

[46] Scholz A (2011). Angiopoietin-2 promotes myeloid cell infiltration in a β_2_-integrin-dependent manner. Blood.

[47] Sinnathamby T, Yun J, Clavet-Lanthier MÉ, Cheong C, Sirois MG (2015). VEGF and angiopoietins promote inflammatory cell recruitment and mature blood vessel formation in murine sponge/Matrigel model. J Cell Biochem.

[48] Lemieux C, Maliba R, Favier J, Théorêt JF, Merhi Y, Sirois MG (2005). Angiopoietins can directly activate endothelial cells and neutrophils to promote proinflammatory responses. Blood.

[49] Kim J (2019). Tie2 activation promotes choriocapillary regeneration for alleviating neovascular age-related macular degeneration. Sci Adv.

[50] Bruttger J (2015). Genetic cell ablation reveals clusters of local self-renewing microglia in the mammalian central nervous system. Immunity.

[51] Russo MV, Latour LL, McGavern DB (2018). Distinct myeloid cell subsets promote meningeal remodeling and vascular repair after mild traumatic brain injury. Nat Immunol.

[52] Kuchroo VK, Martin CA, Greer JM, Ju ST, Sobel RA, Dorf ME (1993). Cytokines and adhesion molecules contribute to the ability of myelin proteolipid protein-specific T cell clones to mediate experimental allergic encephalomyelitis. J Immunol.

[53] Petersen MA, Ryu JK, Akassoglou K (2018). Fibrinogen in neurological diseases: mechanisms, imaging and therapeutics. Nat Rev Neurosci.

[54] Ryu JK (2018). Fibrin-targeting immunotherapy protects against neuroinflammation and neurodegeneration. Nat Immunol.

[55] Jiang H, Zhang F, Yang J, Han S (2014). Angiopoietin-1 ameliorates inflammation-induced vascular leakage and improves functional impairment in a rat model of acute experimental autoimmune encephalomyelitis. Exp Neurol.

[56] Sun JF (2005). Microvascular patterning is controlled by fine-tuning the Akt signal. Proc Natl Acad Sci U S A.

[57] Stromnes IM, Goverman JM (2006). Active induction of experimental allergic encephalomyelitis. Nat Protoc.

[58] Flach AC (2016). Autoantibody-boosted T-cell reactivation in the target organ triggers manifestation of autoimmune CNS disease. Proc Natl Acad Sci U S A.

[59] Schläger C, Litke T, Flügel A, Odoardi F (2016). In vivo visualization of (auto)immune processes in the central nervous system of rodents. Methods Mol Biol.

[60] Warnick RE, Fike JR, Chan PH, Anderson DK, Ross GY, Gutin PH (1995). Measurement of vascular permeability in spinal cord using Evans Blue spectrophotometry and correction for turbidity. J Neurosci Methods.

[61] Georgiadou M (2017). AMPK negatively regulates tensin-dependent integrin activity. J Cell Biol.

[62] Lilja J (2017). SHANK proteins limit integrin activation by directly interacting with Rap1 and R-Ras. Nat Cell Biol.

[63] Butler A, Hoffman P, Smibert P, Papalexi E, Satija R (2018). Integrating single-cell transcriptomic data across different conditions, technologies, and species. Nat Biotechnol.

[64] Huang da W, Sherman BT, Lempicki RA (2009). Systematic and integrative analysis of large gene lists using DAVID bioinformatics resources. Nat Protoc.

[65] Huang da W, Sherman BT, Lempicki RA (2009). Bioinformatics enrichment tools: paths toward the comprehensive functional analysis of large gene lists. Nucleic Acids Res.

[66] Zudaire E, Gambardella L, Kurcz C, Vermeren S (2011). A computational tool for quantitative analysis of vascular networks. PLoS One.

