# Supplementary material for: Angiopoietin-2 blockade ameliorates autoimmune neuroinflammation by inhibiting leukocyte recruitment into the CNS
Source: J Clin Invest. 2020 Mar 9;130(4):1977–90. doi: 10.1172/JCI130308 (PMC7108925; doi:10.1172/JCI130308)
Supplement: Supplemental data [file jci-130-130308-s318.pdf]

Supplemental material

Angiopoietin-2 blockade ameliorates autoimmune neuroinflammation by inhibiting leukocyte recruitment into the CNS

Zhilin Li,[1] Emilia A. Korhonen,[1] Arianna Merlini,[2] Judith Strauss,[2] Eleonoora Wihuri,[1] Harri Nurmi,[1] Salli Antila,[1] Jennifer Paech,[1] Urban Deutsch,[3] Britta Engelhardt,[3] Sudhakar Chintharlapalli,[4] Gou Young Koh,[5,6] Alexander Flügel,[2] Kari Alitalo[1,7]

[1]Wihuri Research Institute and Translational Cancer Medicine Program, Faculty of Medicine, University of Helsinki, Helsinki, Finland

[2]Institute for Neuroimmunology and Multiple Sclerosis Research, University Medical Centre Göttingen, Göttingen, Germany

[3]Theodor Kocher Institute, University of Bern, Bern, Switzerland

[4]Oncology Research; Lilly Research Laboratories, Eli Lilly and Company, Indianapolis, Indiana, USA

[5]Center for Vascular Research, Institute for Basic Science, Daejeon, South Korea

[6]Graduate School of Medical Science and Engineering, Korea Advanced Institute of Science and Technology (KAIST), Daejeon, South Korea

[7]Helsinki Institute of Life Science, University of Helsinki, Helsinki, Finland

Authorship note: ZL and EAK contributed equally to this work.

Address correspondence to Kari Alitalo, Wihuri Research Institute and Translational Cancer Medicine Program, Biomedicum Helsinki, 00014 University of Helsinki, Haartmaninkatu 8, Helsinki, Finland.

Phone: +358 2 941 25511; Email: kari.alitalo@helsinki.fi

Supplemental Data

Supplemental Table 1. Genes regulated by both Ang2 blockade and Ang2 overexpression in scRNA-seq.

Cluster name	List of overlapping genes
**Microglia**	*Aldoa, Anxa5, AW112010, Ccl5, Cd74, Cst7, Cxcl9, Fabp5, Fth1, Ftl1, Gpnmb, H2-Aa, H2-Ab1, H2-Eb1, H2-K1, H2-Q7, Hp, Il18bp, Il2rg, Ly6i, Pla2g7, Saa3, Thbs1*
**Macrophages**	*Acp5, Cd63, Cd68, Ctsb, Ctsd, Ctsl, Ecm1, Fabp5, Gpnmb, Grn, Hexa, Ms4a7, Rgs1, Saa3*
**Capillary-venous ECs**	*Ackr1, AW112010, B2m, Cadm3, Cd82, Clec14a, Ctla2a, Ecm1, Gbp2, Gbp3, Gbp4, Gbp5, Gbp7, H2-D1, H2-K1, H2-Q6, H2-Q7, H2-T22, Igtp, Iigp1, Irgm1, Lyz2, Prcp, Psmb8, Psmb9, Rnase4, Saa3, Stat1, Tap1, Tapbp, Tmsb10, Upp1, Vcam1, Vim, Vwf*

Supplemental Table 2. List of primer sequences used for RT-qPCR analysis.

Gene	Forward primer sequence (5’-3’)	Reverse primer sequence (5’-3’)
Itga4	AATCCAAACCAGACCTGCGA	TTTTCCACCTGTGCCCACAA
Itgb1	CGGACGCTGCGAAAAGATGA	CATTCTCCGCAAGATTTGGCA
Ifng	ATGAACGCTACACACTGCATC	CCATCCTTTTGCCAGTTCCTC
Tnf	CCAGTGTGGGAAGCTGTCTT	AAGCAAAAGAGGAGGCAACA
Il4	GGTCTCAACCCCCAGCTAGT	GCCGATGATCTCTCTCAAGTGAT
Il17a	TTTAACTCCCTTGGCGCAAAA	CTTTCCCTCCGCATTGACAC
H2-Aa	TAGCAAGTCAGTCGCAGACG	TCTCAGGTTCCCAGTGTTTCAG
H2-Ab1	CAGGAGTCAGAAAGGACCTCG	ACTGGCAGTCAGGAATTCGG
H2-D1	ACCTGCAGTTCGCCTATGAAG	TAATGCTCTGCAGCACCACTCT
H2-Eb1	GTCGAGTGGAAAGCACAATCC	AGTAGATGAACAGCCCCGCT
H2-K1	CCGCGGACGCTGGATATAA	GACACGGCGGTGACGAAATA
H2-Q7	GGGAGCGGGTTGTAAAGTCC	ACCAGCAAGAGCAGCATTGT
Fabp5	GGCAACAACATCACGGTCAA	GGTGCAGACCGTCTCAGTTTT
Lgals3	AACCCAACGCAAACAGGATT	AGCGGGGGTTAAAGTGGAAG
Tyrobp	GAGTGACACTTTCCCAAGATGC	CCTTGACCTCGGGAGACCA
Icam1	GTGATGCTCAGGTATCCATCCA	CACAGTTCTCAAAGCACAGCG
Pecam1	CTGCCAGTCCGAAAATGGAAC	CTTCATCCACTGGGGCTATC
Rplp0	GGACCGCCTGGTTCTCCTAT	ACGATGTCACTCCAACGAGG
Hprt	TTGCTCGAGATGTCATGAAGGA	AGCAGGTCAGCAAAGAACTTATAG

Supplemental Figure 1

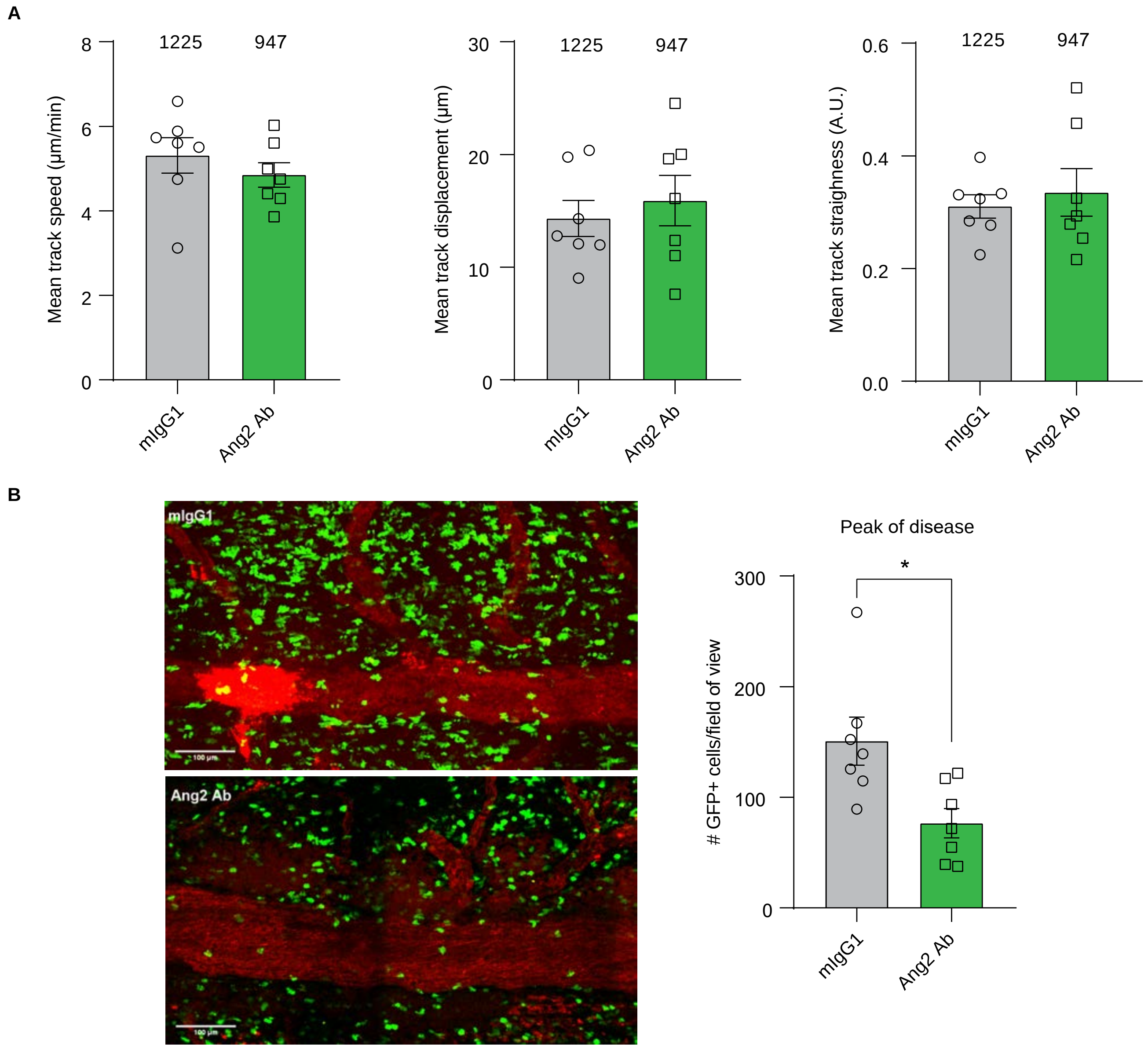

Supplemental Figure 1. Prophylactic Ang2 blockade attenuates T cell infiltration but does not affect T cell motility in adoptive transfer EAE. (**A**) Motility characteristics of $MOG_{35\text{-}55}$ reactive T cells in SC areas recorded by intravital two-photon microscopy over a period of 30 min (n = 7 per group). Each dot represents data obtained from one mouse. The total number of analyzed cells is indicated. (**B**) Representative two-photon microscopic images and quantification showing the pattern of infiltration of $MOG_{35\text{-}55}$ reactive T cells in the SC leptomeninges and subpial layers at the peak of the disease in mice treated with mIgG1 or Ang2 antibody (n = 7 per group). Scale bar, 100 µm. Green: $MOG_{35\text{-}55}$ reactive T cells; Red: leptomeningeal vessels. Mean ± SEM, two-tailed Student's *t*-test. * $P < 0.05$.

Supplemental Figure 2

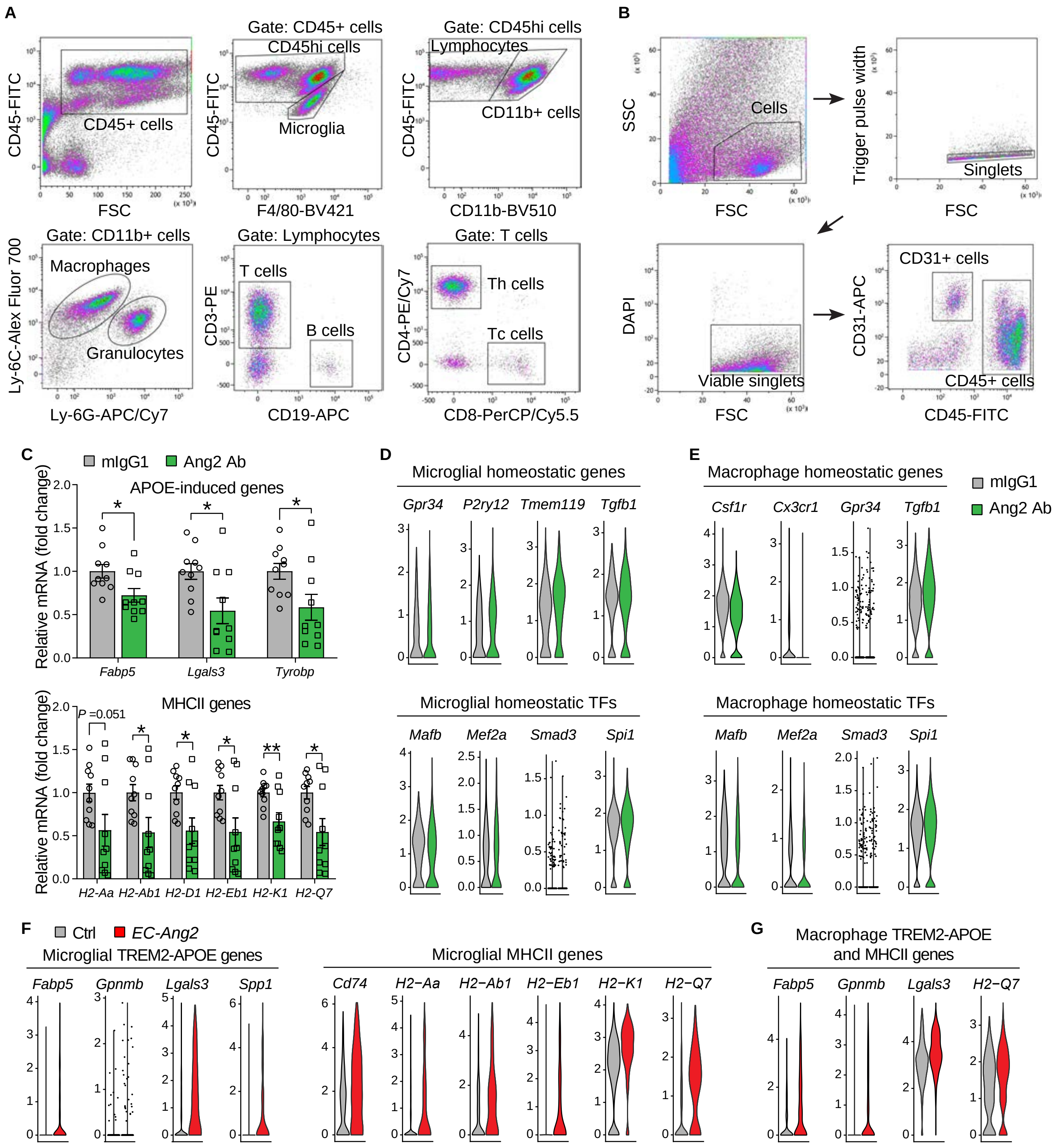

Supplemental Figure 2. Prophylactic Ang2 blockade dampens immune activation but does not affect homeostatic genes of myeloid cells in the SCs of EAE mice. (**A**) Flow cytometric gating strategy for the analysis of immune cells enriched from the SCs. (**B**) Flow cytometric gating strategy for the sorting of immune cells and ECs from the SCs. (**C**) RT-qPCR quantification of APOE-induced and MHCII-associated genes in the SCs of mIgG1- vs Ang2 antibody-treated EAE mice at 14 dpi (n = 10 per group). (**D** and **E**) Violin plots showing expression of microglial and macrophage homeostatic genes and transcription factors (TFs) in the SCs of mIgG1- vs Ang2 antibody-treated mice at 14 dpi. (**F** and **G**) Violin plots showing significantly (adjusted P < 0.05) upregulated microglial and macrophage APOE-induced and MHCII-associated genes in the SCs of Ctrl vs *EC-Ang2* mice at 12 dpi. Mean ± SEM, two-tailed Student's t-test (**C**). * P < 0.05, ** P < 0.01.

Supplemental Figure 3

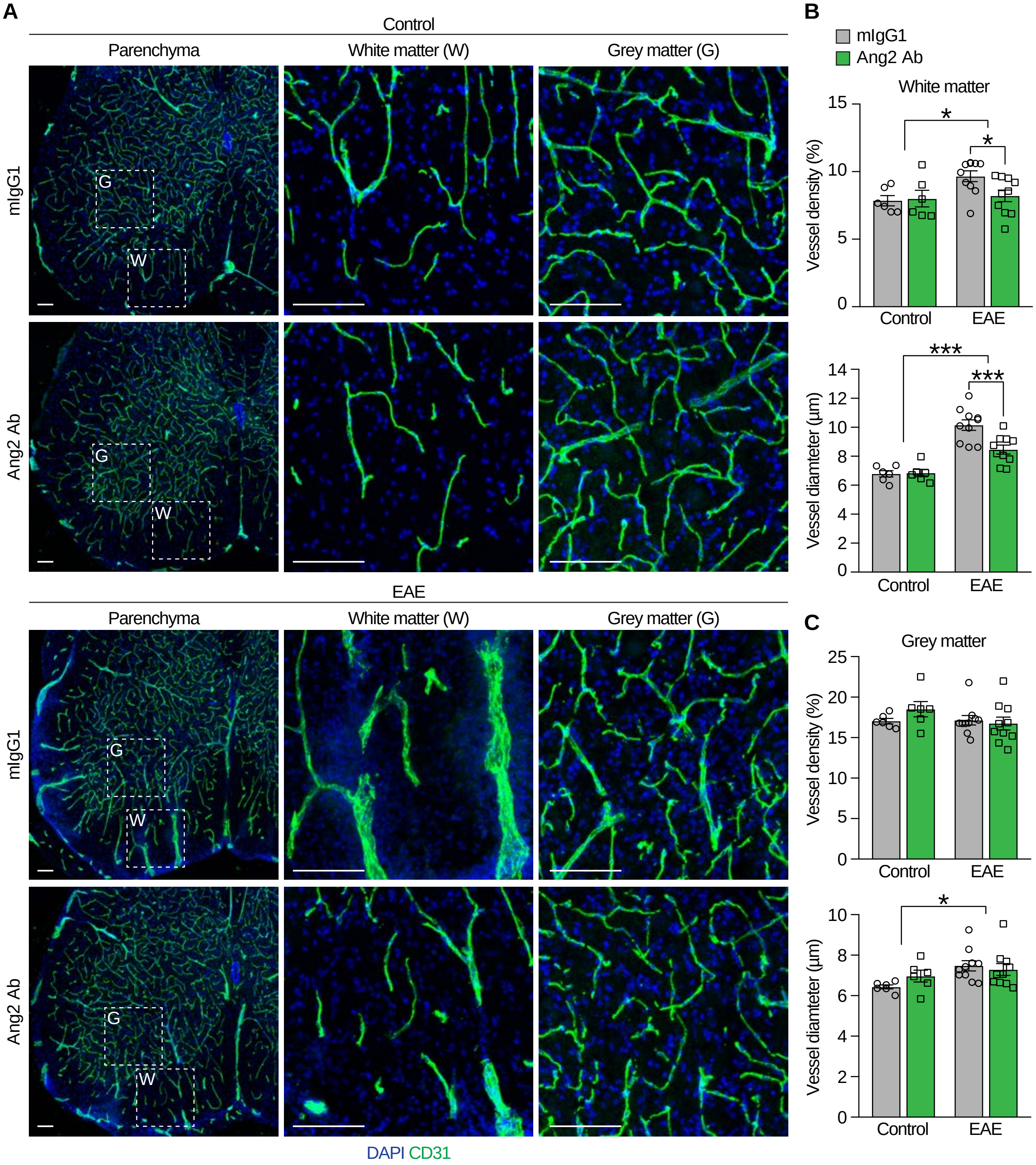

Supplemental Figure 3. Prophylactic Ang2 blockade inhibits vascular remodeling in the SCs of EAE mice. (**A**) Representative images of CD31$^+$ blood vessels in the SCs of mIgG1- vs Ang2 antibody-treated control (n = 6 per group) and EAE (n = 10 per group) mice (14 days). Scale bar, 100 µm. (**B**) Quantification of vessel density and diameter in the SC white matter (W). (**C**) Quantification of vessel density and diameter in the SC grey matter (G). Mean ± SEM, two-way ANOVA with Bonferroni's post-hoc test for multiple comparisons. * $P < 0.05$, *** $P < 0.001$.

Supplemental Figure 4

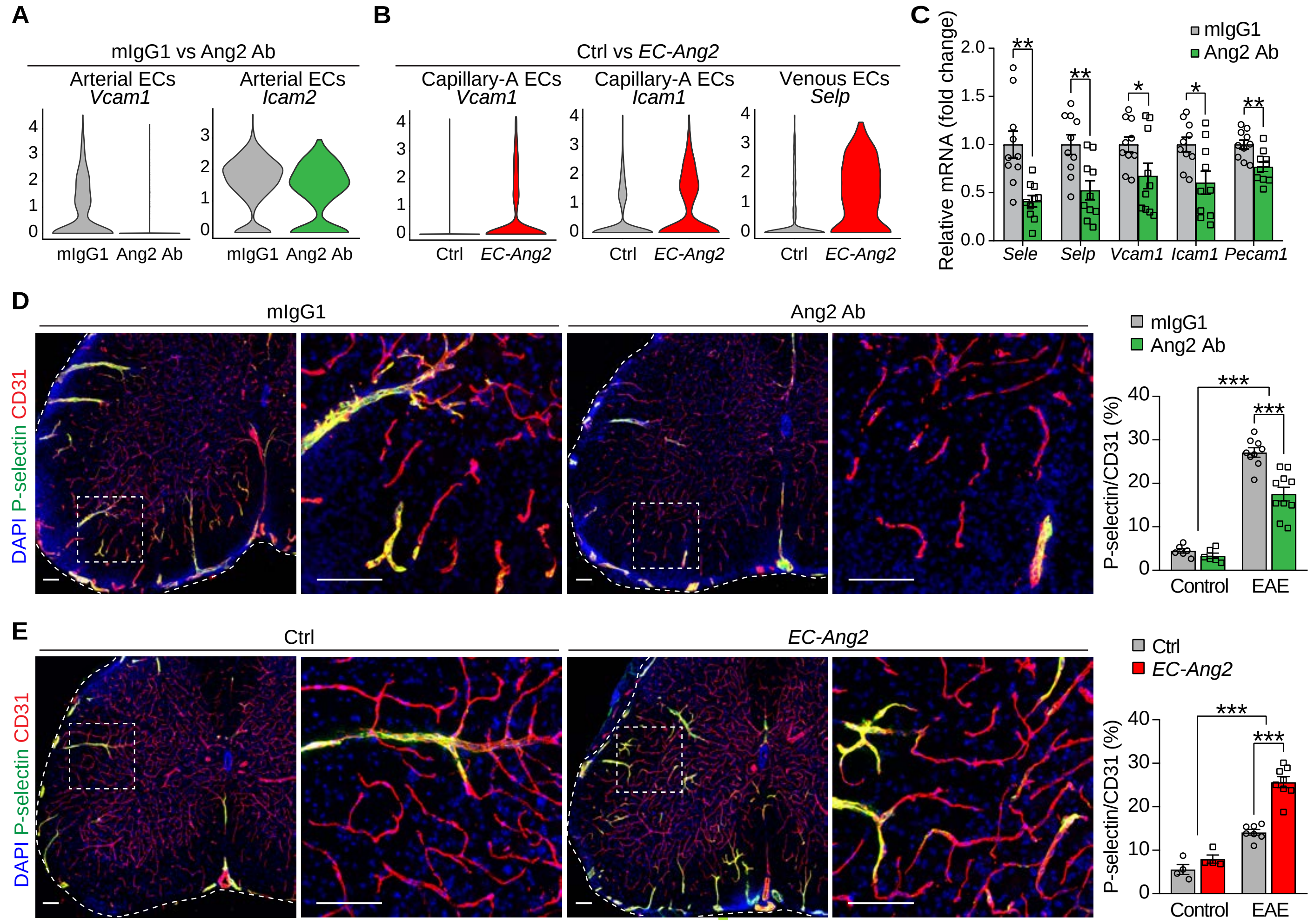

Supplemental Figure 4. Prophylactic Ang2 blockade suppresses vascular inflammation in the SCs of EAE mice. (**A**) Violin plots showing mRNA expression of significantly (adjusted $P < 0.05$) downregulated *Vcam1* and *Icam2* in the SC arterial ECs of mIgG1- vs Ang2 antibody-treated EAE mice at 14 dpi. (**B**) Violin plots showing mRNA expression of significantly (adjusted $P < 0.05$) upregulated *Vcam1* and *Icam1* in the capillary-arterial ECs, and *Selp* in the venous ECs in the SCs of Ctrl- vs *EC-Ang2* EAE mice at 12 dpi. (**C**) RT-qPCR quantification of mRNA levels of leukocyte adhesion molecules (*Sele*, *Selp*, *Vcam1*, *Icam1* and *Pecam1*) in the SCs of mIgG1- vs Ang2 antibody-treated EAE mice at 14 dpi (n = 10 per group). (**D** and **E**) Representative images (EAE) and quantification of P-Selectin in the SC blood vessels of mIgG1- vs Ang2 antibody-treated control (n = 6 per group) and EAE (mIgG1: n = 9; Ang2 Ab: n = 10) mice at 14 dpi, and Ctrl vs *EC-Ang2* control (Ctrl: n = 4; *EC-Ang2*: n = 4) and EAE (Ctrl: n = 7; *EC-Ang2*: n = 8) mice at 12dpi. Scale bar, 100 µm. Mean ± SEM, two-tailed Student's *t*-test (**C**), two-way ANOVA with Bonferroni's post-hoc test for multiple comparisons (**D** and **E**). * $P < 0.05$, ** $P < 0.01$, *** $P < 0.001$.
